# Spatiotemporal Trends Spanning Three Decades Show Toxic Levels of Chemical Contaminants in Marine Mammals

**DOI:** 10.1021/acs.est.3c01881

**Published:** 2023-11-27

**Authors:** Rosie S. Williams, Andrew Brownlow, Andrew Baillie, Jonathan L. Barber, James Barnett, Nicholas J. Davison, Robert Deaville, Mariel ten Doeschate, Sinéad Murphy, Rod Penrose, Matthew Perkins, Simon Spiro, Ruth Williams, Paul D. Jepson, David J. Curnick, Susan Jobling

**Affiliations:** † Institute of Zoology, Zoological Society of London, Regent’s Park, London NW1 4RY, United Kingdom; ‡ School of Biodiversity One Health and Veterinary Medicine, College of Medical, Veterinary & Life Sciences, University of Glasgow, Glasgow G12 8QQ, United Kingdom; § Department of Life Sciences, Institute of Health, Medicine and Environments, Brunel University London, Uxbridge UB8 3PH, United Kingdom; ∥ Centre for Environment, Fisheries and Aquaculture Science (Cefas), Pakefield Road, Lowestoft NR33 0HT, United Kingdom; ⊥ Environment and Sustainability Institute, University of Exeter, Penryn Campus, Falmouth, Cornwall TR10 9FE, United Kingdom; # The Natural History Museum, Cromwell Road, London SW7 5BD, United Kingdom; ∇ Marine Environmental Monitoring, Penwalk, Llechryd, Cardigan, Ceredigion SA43 2PS, United Kingdom; ○ Cornwall Wildlife Trust, Truro, Cornwall TR4 9DJ, United Kingdom; ◆ Marine and Freshwater Research Centre, Department of Natural Science, School of Science and Computing, Galway-Mayo Institute of Technology, Galway H91 T8NW, Ireland; ▼ Department of Genetics, Evolution and Environment, University College London, Darwin Building, 99-105 Gower Street, London WC1E 6BT, United Kingdom

**Keywords:** marine mammals, persistent organic pollutants, temporal trend, ecotoxicology, cetaceans, PCBs, POPs, polychlorinated biphenyls

## Abstract

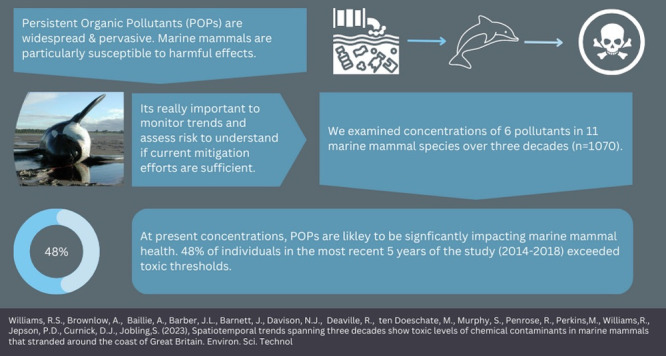

Despite their ban and restriction under the 2001 Stockholm Convention, persistent organic pollutants (POPs) are still widespread and pervasive in the environment. Releases of these toxic and bioaccumulative chemicals are ongoing, and their contribution to population declines of marine mammals is of global concern. To safeguard their survival, it is of paramount importance to understand the effectiveness of mitigation measures. Using one of the world’s largest marine mammals strandings data sets, we combine published and unpublished data to examine pollutant concentrations in 11 species that stranded along the coast of Great Britain to quantify spatiotemporal trends over three decades and identify species and regions where pollutants pose the greatest threat. We find that although levels of pollutants have decreased overall, there is significant spatial and taxonomic heterogeneity such that pollutants remain a threat to biodiversity in several species and regions. Of individuals sampled within the most recent five years (2014–2018), 48% of individuals exhibited a concentration known to exceed toxic thresholds. Notably, pollutant concentrations are highest in long-lived, apex odontocetes (e.g., killer whales (*Orcinus orca*), bottlenose dolphins (*Tursiops truncatus*), and white-beaked dolphins (*Lagenorhynchus albirostris*)) and were significantly higher in animals that stranded on more industrialized coastlines. At the present concentrations, POPs are likely to be significantly impacting marine mammal health. We conclude that more effective international elimination and mitigation strategies are urgently needed to address this critical issue for the global ocean health.

## Abstract 2

This study investigates concentrations of persistent organic pollutants in 11 species of marine mammal over three decades. We report that although levels have declined there is spatial and taxonomic heterogeneity such that pollutants remain a threat to biodiversity.

## 1 Introduction

Since the 1920s, the increasing global use of thousands of synthetic chemicals in pest and disease control, crop production, and industry has led to unforeseen pervasive and widespread environmental contamination by persistent organic pollutants (POPs). POPs are a group of chemicals that are of grave concern as they are toxic to humans and wildlife, are present in all biota, and have the ability to biomagnify and bioaccumulate throughout food webs due to their persistence and lack of biotransformation.^1,2^ They have been shown to cause considerable harm (e.g., deleterious effects on immunity, reproduction, and development) to wildlife populations and humans.^3,4^ The impact of POPs in the marine environment is most acute in long-lived, top predators including several species of marine mammals. Several countries limited the use of some POPs (e.g., PCBs and some organochlorine pesticides) in the 1970s and their use has been heavily restricted in Europe since 1985. This culminated in the Stockholm Convention (a multilateral treaty to protect human health and the environment from POPs) which came into force in 2004 and prohibited the production and use of several POPs in more than 152 countries.^5^ Despite the initial success of national and international regulatory agreements, tissue concentrations remain at hazardous levels in many wildlife species as a consequence of their persistent nature, continued use in some regions and a failure to prevent environmental releases.^6−9^ At present rates of elimination, it is expected that several parties to the Convention will fail to meet their forthcoming commitments to eliminate the use of PCBs in equipment by 2025.^7,10,11^ In addition, secondary releases of POPs into the environment may increase as a consequence of climate change due to changes in the fate and behavior of POPs^12^ and releases from historic coastal landfills (built before the introduction of stringent environmental regulation) caused by flooding, erosion and sea level rise.^13^ It is estimated that there are 10 000 historic landfill sites on European coasts with the potential to release pollutants, such as PCBs, directly into the marine environment.^13^ There exists now a timely opportunity to evaluate the vulnerability of long-lived apex predators ahead of the twelfth meeting of the Conference of the Parties (COP) to the Stockholm Convention in 2025..

Marine mammals are mobile with many species occupying higher trophic levels and are therefore considered effective sentinels of ocean health.^14^ Organochlorine pesticides (OCPs) and polychlorinated biphenyls (PCBs), in particular, have been shown to cause suppression of the immune and reproduction systems in mammalian species,^3,6,15,16^ and are thought to be contributing to population declines and lower recruitment observed in several European marine mammal populations.^17^ Populations that inhabit heavily contaminated, semi-industrial marine habitats such as the North-East Atlantic, Mediterranean and Gibraltarian Strait, are thought to be most vulnerable.^17,18^ Marine mammals that strand around Great Britain inhabit seas that surround a heavily industrialized area where environmental releases of legacy pollutants still take place and large stockpiles of pollutants are yet to be destroyed.^7^ This area is characteristic of many other regions around the globe where marine mammals live in close proximity to highly industrialized coasts and therefore, face a number of anthropogenic threats alongside chemical pollution (e.g., bycatch, acoustic disturbance, prey depletion).^19,20^ The legacy of past manufacture and release of persistent contaminants, combined with continued release from historically contaminated sites, presents a lingering risk to vulnerable species.^7,21^ Understanding more about pollutant concentrations in these regions is essential to be able to rigorously and robustly determine whether current elimination and mitigation actions are sufficient and to aid the development of effective conservation and management strategies for marine ecosystems.

Here, we explore pollutant concentrations in marine mammals that stranded along the coast of Great Britain, using one of the largest marine mammal strandings data sets available globally, consisting of 11 species that stranded over three decades. The aims of our study were to (1) examine the pollutant concentrations of six classes of persistent organic pollutants using novel and previously published blubber pollutant data collected from 1070 individuals (further details on which data have been previously published are provided in the methods and Table S11), (2) investigate the influence of spatiotemporal factors and interspecific variation on pollutant concentrations, and (3) quantify collective risks and relative risks, to the immune and endocrine systems, for each class of pollutant.

## 2 Methodology

### 2.1 Sampling

**Figure 1:**
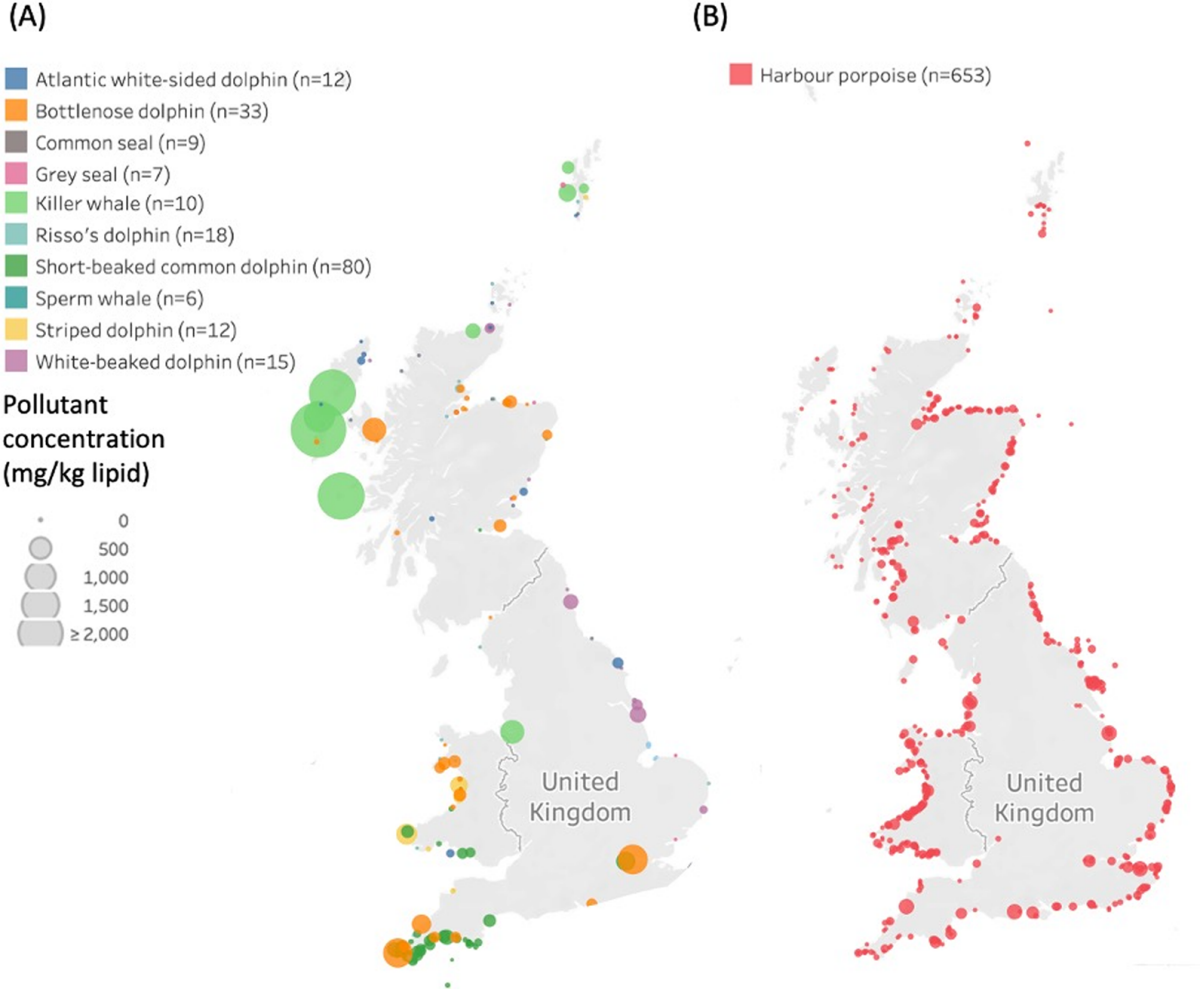
Geographic locations of the stranded individuals that were analyzed to obtain pollutant blubber concentrations (HCB, Dieldrin, ∑DDTs, ∑CBs, ∑HCHs). PBDEs were excluded from the summed totals as tissue concentrations were not available for some of the species. The colors of the dots represent the different species, and the raw data are sized by the summed blubber concentrations of pollutants. (A) All species, excluding harbor porpoises. (B) Harbour porpoises.

Necropsies were carried out between 1990 and 2018, by the United Kingdom Cetacean Strandings Investigation Programme (CSIP), on over 4000 carcasses according to standard procedures for marine mammals.^22^ Carcasses were then prioritized for pollutant analysis according to their state of decomposition using a standardized classification system for marine mammals.^23^ Carcasses with a decomposition code greater than or equal to four (advanced decomposition) were excluded from the analysis. This led to a sample size of 1070 individuals: Atlantic white-sided dolphins (*Lagenorhynchus acutus*) (*n* = 22); bottlenose dolphins (*Tursiops truncatus*) (*n* = 63); common seals (*Phoca vitulina*) (*n* = 16); gray seals (*Halichoerus grypus*) (*n* = 21); harbor porpoises (*Phocoena phocoena*) (*n* = 731); killer whales (*Orcinus orca*) (*n* = 15); Risso’s dolphins (*Grampus griseus*) (*n* = 26); short-beaked common dolphins (*Delphinus delphis*) (*n* = 124); striped dolphins (*Stenella coeruleoalba*) (*n* = 22); sperm whales (*Physeter macrocephalus*) (*n* = 6); and white-beaked dolphins (*Lagenorhynchus albirostris*) (*n* = 24) (Figure 1). Data on the sex, developmental stage, and health status are available from the lead author on request. Of the carcasses analyzed for pollutants, 87% were classified as extremely fresh or slightly decomposed (codes 2a and 2b). Carcasses were prioritized in this way to minimize the impact of changes in pollutant concentrations and lipid dispersion that are associated with decomposition.^24^ We ensured that the individuals analyzed were a representative sample of the strandings that occurred over the study period by testing for statistical differences in the proportions of cause of death, sex, age, body weight, length and seasonality between the contaminants data set and the complete strandings data set (*n* > 15 000).

### 2.2 Pollutant Analyses

Blubber concentrations of six pollutant classes were determined across all 11 species of marine mammals examined. The six pollutant classes have widespread applications either as crop treatments, (1) isomers of dichlorodiphenyltrichloroethane (DDTs), (2) hexachlorocyclohexanes (HCHs), (3) hexachlorobenzene (HCB), (4) dieldrin, or as industrial chemicals, (5) PCB congeners and (6) flame retardants (polybrominated diphenyl ethers (PBDEs). All groups are known to be toxic to marine life (Table S1).

To conduct this analysis, we have combined data from previous studies^6,17,25,26^ with newly generated, unpublished data to conduct comparative analysis across multiple species and multiple pollutant classes. Of the 11 species included in this study, data have been published for three of the species, and the data for the remaining eight species are unpublished. Across the six pollutants and 11 species, a total of 5260 pollutant concentrations were included in this study (*n* = 2006 unpublished, *n* = 3254 previously published). Further details on which data have been previously published are provided in Table S11. For each individual, a full thickness blubber sample was taken (from the dorsolateral region close to the insertion of the dorsal fin in cetaceans and from the ventral thorax in pinnipeds), wrapped in catering grade foil and preserved at −20 °C using established protocols.^22^ Pollutant concentrations were determined (on a mg kg^–1^ wet weight basis) at the Cefas (Centre for Environment, Fisheries and Aquaculture Science) laboratory (Lowestoft) using methods that follow the recommendations of the International Council for the Exploration of the Sea (ICES) and validated under the QUASIMEME laboratory proficiency scheme.^27^ Concentrations of DDTs, HCHs, HCB, dieldrin, and PCBs were measured using gas chromatography electron capture detection (GC-ECD).^27^ Concentrations of PBDEs were determined using gas chromatography with detection by electron capture negative ion mass spectrometry (GC-ECNIMS), monitoring the bromine ions at 79 and 91 Da.^28^

For quality assurance and quality control the CEFAS laboratory (Lowestoft) participates biannually in the QUASIMEME (Quality Assurance of Information for Marine Environmental Monitoring in Europe) proficiency testing scheme. All analyses were carried out under full analytical quality control procedures, which included the analysis of a blank sample and the analysis of a certified reference material with every batch of 10 samples to assess the performance of the methods. Blanks for individual pollutants were always below the limit of quantitation. Where the levels of target analytes were beyond the range of the instrument calibration, we diluted and reanalyzed the extract. We used the reference material BCR349 (cod liver oil; European Bureau of Community reference) and for each compound and we plotted the reference material results as Shewhart quality control charts. The charts were created previously from repeated analysis of the reference material using the North West Analytical Quality Analyst software (Northwest Analytical Inc., USA). The warning and control limits for the charts were defined as 2σ and 3σ, 2× and 3× the standard deviation from the mean for each compound. For each of the samples analyzed the certified reference materials were within the limits set by the control charts. Therefore, all results were deemed to be valid. The expanded uncertainty MU (calculated as 2*standard deviation of the control charts for the BCR349 reference material from the last 10 years) for the ICES7 PCBs ranges from 11.9% for CB153 to 17.9% for CB28, which is well within the requirement to be <50%. The percentage of nondetects for the 25 PCB congeners, 3 HCH isomers, 3 DDT isomers, 11 BDE congeners, and HCB are shown in Table S12.

In cases where concentrations were below the limit of quantification, the concentrations were set at half the limit.^25^ Pollutants and their respective congeners/isomers investigated here cover a wide range of physical and chemical properties, include congeners that can be compared with other studies and measurement standards (e.g., seven PCBs prioritized for international monitoring by ICES) and have a wide range of applications. The measured pollutants also vary in terms of historical use, persistence and toxicities (Table S1).^29^

### 2.3 Statistical Analyses

All statistical analyses were carried out using the statistical computer program R (version 4.0).^30^ To investigate any differences due to age class, individuals were categorized into three age and sex classes (juveniles, adult females, and adult males) according to body length and sexual maturity as per Jepson et al..^17^ Sexual maturity was assessed using gonadal analysis, including assessments of spermatogenesis and ovarian folliculogenesis.^26^ As part of the cetacean pathological investigations, dorsal, ventral, and lateral blubber thickness were measured and the mean thickness for each individual was calculated. A linear regression model was fitted to the log of the mean blubber thickness, with species as the predictor variable, and the model residuals were used as a proxy for body condition. The model residuals were plotted against cause of death and body weight to length ratios to verify that this approach was suitable.

Initial analyses of blubber pollutant concentrations covered all 11 species (*n* = 1070) (Figure 1). Comparisons between species were made by calculating the mean blubber concentrations for each pollutant. In addition, differences in pollutant concentrations and relative abundances were assessed using Kruskal–Wallis and posthoc Dunn’s tests due to the non-normality of some of the pollutants (n = 1070). Differences in PCB contamination profiles were investigated by examining patterns in variation for individual congeners and congener groupings using Kruskal–Wallis and posthoc Dunn’s test to assess differences between species. Congeners were grouped according to their degree of chlorination and their dioxin-like properties. Similarly, for PBDEs, variation was assessed for individual congeners and for congeners grouped according to their degree of bromination.

#### 2.3.1 Spatiotemporal Variation

To investigate the factors that influence pollutant concentrations, linear regression models were fitted to selected variables that could explain the variability in the data.^31,32^ Killer whales, sperm whales, gray seals, and common seals (*n* = 58) were excluded from the spatiotemporal trends analyses because of their low sample size and high variance (Table S2). The flame retardants (PBDEs) were also excluded from statistical modeling because analyses for these pollutants were only carried out on some of the species included in the models. However, summary data (e.g., mean, max, and minimum concentrations for each species) were included as well as information on the mean relative abundances of higher and lower brominated PBDEs. PBDEs were also included in the comparative risk assessment, outlined below. Prior to model fitting, extensive data exploration was carried out to test for collinearity between variables and to remove individuals with missing biological data or those for which there were incomplete results for any of the five pollutant classes included in the models. This resulted in a total sample size of 745 (Table S2).

Models were fitted to the summed and individual concentrations of pollutants (PCBs, DDTs, HCHs, dieldrin, and HCB). For each model, the log transformed pollutant concentration was the response variable. The predictor variables included in the full models were selected according to the biological rationale that they could influence pollutant concentrations. These were year of stranding, age class, sex, latitude, longitude, species, and the residuals of the log-model fitted to blubber thickness (as a proxy for body condition), including two-way interaction terms between age class and sex and a four-way interaction term between latitude, longitude, species, and year of stranding.^33^

For each model, all possible variable combinations were tested to obtain several candidate models. Final predictions were obtained by averaging the set of plausible models (Δ Akaike’s Information Criterion (AIC) < 4) from the candidate models.^34,35^ The models were validated by assessing the normality of the residuals, plotting them against selected variables, and assessing the variance. The model coefficients were used to predict spatiotemporal trends in concentrations; this included separate analyses of trends in OSPAR (Oslo and Paris Conventions) contaminants assessment areas. Pollutants in marine mammals are not typically assessed as part of OSPAR (however, a pilot assessment was carried out in 2022 at the OSPAR region level^36^); therefore, the contaminant assessment areas defined by OSPAR to investigate pollutant trends in fish and shellfish were used for this analysis (Figure 3B).^37^ The OSPAR contaminant areas assessed were the Irish & Scottish West Coast, the Irish Sea, the Celtic Sea, the Channel, the Southern North Sea, and the Northern North Sea.^37^

#### 2.3.2 Risk Assessment

To assess the toxicity, we calculated the proportion of individuals with blubber concentrations that exceed selected published toxicity thresholds (Table 1). We also plotted histograms of the log-transformed tissue concentrations from the first five years of the study (1990–1994) and the most recent five years of the study (2014–2018) to compare changes in the distribution of pollutant concentrations in relation to toxicity thresholds over time. This analysis was only carried out for three pollutants (PCBs, PBDEs, and *pp*′-DDE) as toxicity thresholds for marine mammals have not been derived for the other pollutants. As there are several published thresholds for PCBs, we used the threshold derived by Kannan et al., who incorporated results from several studies on captive and free-ranging marine mammals (Table 1).^38−40^

**Table 1:** Published Threshold Tissue Concentrations of PCBs, PBDEs, and pp′DDE in Marine Mammals^a^

Pollutant	Threshold	Species	End point	Reference
PCBs	9 mg/kg lipid	Marine Mammals	Hepatic vitamin A, thyroid hormone concentration, natural killer cell activity, lymphocyte response	Jepson et al., 2016; Kannan et al., 2000^38,41^
	5.42 mg/kg lipid	Cetaceans	Decreased lymphocyte proliferation (Effective concentrations giving a 1% response (EC1))	Desforges et al., 2016^42^
	0.14 mg/kg lipid	Bottlenose dolphins	Decreased lymphocyte proliferation (effective concentrations giving a 1% response (EC1))	Desforges et al., 2016^42^
	7.1–15.1 mg/L ww	Harbour seals	Pooled blood samples from controlled groups for increased lymphocytes, granulocytes, and basophils and decreased monocytes	de Swart et al. 1994, Reijnders 1988^40,43^
	1.3 mg/kg lipid	Harbour seals	Several biomarkers (e.g., plasma retinol and AhR expression) and immune function end points	Mos et al., 2010^44^
	41 mg/kg lipid	Baltic ringed seals	Pathological changes in seal uteri	Helle et al., 1976^45^
	1.6 mg/kg lipid	Beluga	Disruption of vitamin A and E profiles	Desforges et al., 2013^46^
PBDEs	1.5 mg/kg lipid	Gray Seal Pups	Endocrine disruption	Hall et al., 2003^47^
*pp*′-DDE	1.43 mg/kg lipid	Bottlenose Dolphins	Minimum concentration associated with decreased lymphocyte proliferation	Lahvis et al., 1995^48^

a Details of the end points used to derive the thresholds are also provided. PCB class includes ∑PCB, Aroclors, and individual congeners; the PBDE threshold is in reference to total PBDEs.

To compare the relative risks of exposure from each pollutant class, comparative risk quotients (CRQs) were derived for each pollutant and taxon, using the method outlined by Mos et al.^44^ Sperm whales were excluded from this comparative analysis as PBDEs were not analyzed in this species (*n* = 6). Individuals were also excluded from this analysis if complete pollutant data were unavailable, resulting in a sample size of 858. CRQs are not absolute measures of risk but are relative values that can be used to assess the risk of a pollutant relative to others and allow for pollutants to be prioritized according to risk. Mice and rats were chosen as the reference organisms for toxicity because toxicity reference values (TRVs), which are used to derive CRQs, do not exist for marine mammals but the primary mechanisms of toxicity in persistent organic pollutants have been shown to be similar among mammals.^49^ TRVs for each pollutant were taken from the Agency for Toxic Substances and Disease Registry Toxicological Profiles.^50−55^ TRVs are expressed as daily intakes (mg/kg day) rather than tissue concentrations (mg/kg lipid) however, as the TRVs are being used to assess comparative risk between pollutants rather than absolute risk there is no need to convert the values to tissue concentrations as per Mos et al.^44^ CRQs for each pollutant were derived in relation to endocrine disruption and immunosuppression and calculated by dividing absolute tissue concentrations of a pollutant by its toxicity reference values (TRVs) for no observed adverse effects levels (NOAELs) for oral intakes in mice (eq 1). Where data were not available for mice, the NOAELs in rats were used.^44^ The TRVs used to calculate the CRQs are shown in Table S9. The CRQs were used to calculate the percentage contribution of each pollutant toward toxicity to allow the risk of each pollutant toward endocrine disruption and immunosuppression to be assessed in relation to the pollutants. Relative contributions were calculated by dividing the CRQ for each pollutant by the sum of the CRQs for all of the pollutants.
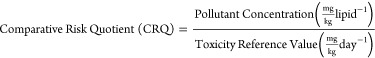
1

## 3 Results

### 3.1 Pollutant Concentrations

**Figure 2:**
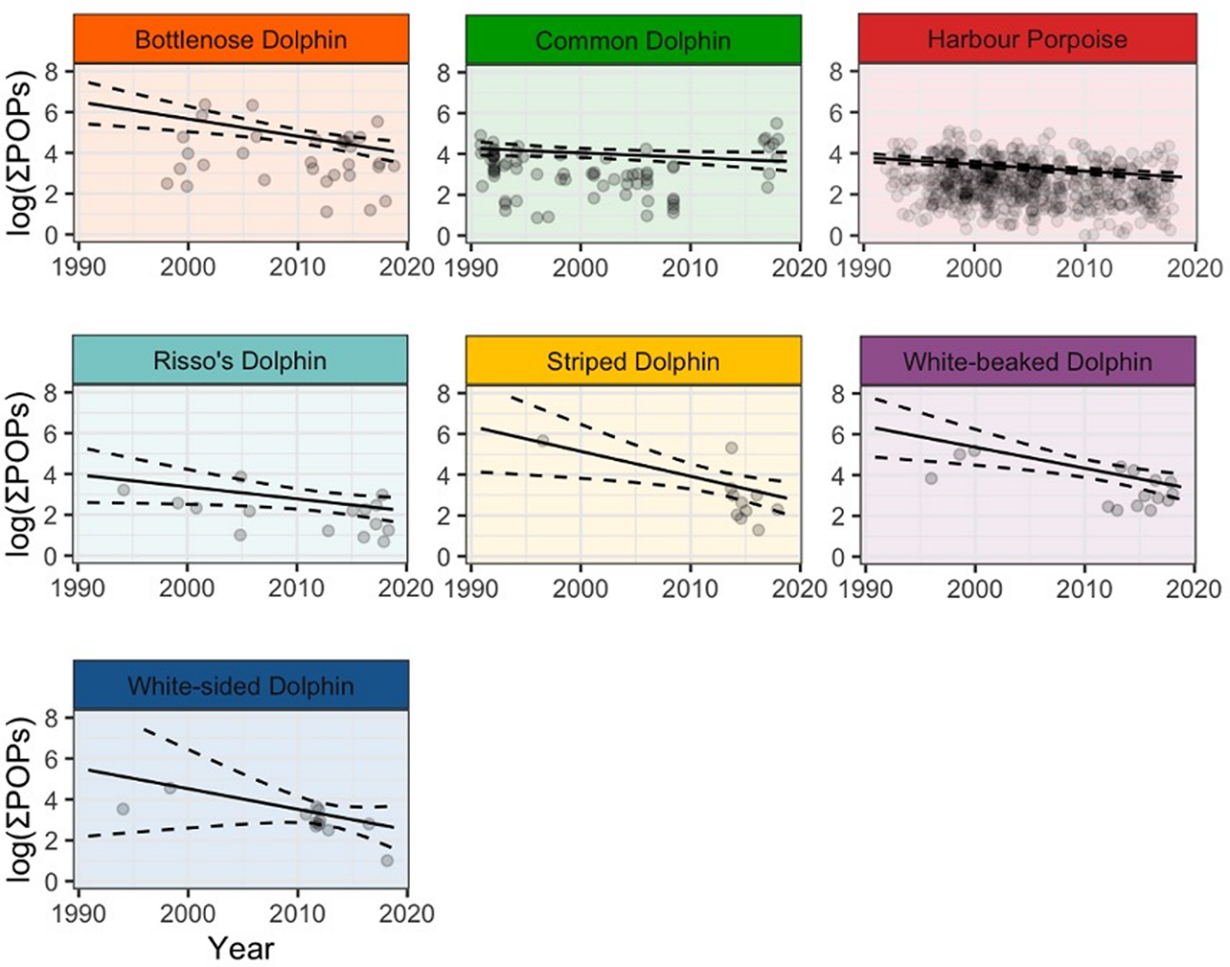
Modeled temporal trend in summed pollutant concentrations (∑POPs) for each species. The solid lines represent the model estimates for each year, and the dashed lines represent 95% confidence intervals (1.96 times the standard error). The dots show the measured pollutant concentrations. Trends lines were calculated using the model coefficients with all variables except for ∑POPs held constant (age group = adult male; mean values were used for blubber thickness, latitude, and longitude). The model coefficients are shown in Table S3. It should be noted that the summed pollutant concentrations were dominated by PCBs and DDTs (Figure S2A).

Analysis of the blubber concentrations of pollutants revealed large inter and intraspecific differences (Table 2, Figures 1 and 2). Killer whales had significantly greater concentrations of summed pollutants (657 mg/kg lipid), 2 orders of magnitude greater than gray seals (*Halichoerus grypus*), which had the lowest mean concentration (8 mg/kg lipid) (Kruskal–Wallis, χ2 (1, *N* = 1070) = 95, *p* < 0.05, Dunn test, *z* = 5.05, *p* = 0) (Table 2 and Table S2). Killer whales had the highest mean concentrations of each individual pollutant; however, differences between species were not always statistically significant (Table 2 and Tables S3–S8).

**Table 2:** Mean, Maximum, and Minimum Blubber Concentrations of Each Persistent Organic Pollutant in Each Species Investigated^a^

	Concentration of pollutant (mg/kg lipid)^b^										
	Atlantic white-sided dolphin (WSD)	Bottlenose dolphin (BND)	Common seal (CS)	Gray seal (GS)	Harbour porpoise (HP)	Killer whale (KW)	Risso’s dolphin (RD)	Short-beaked common dolphin (CD)	Sperm whale (SW)	Striped dolphin (SD)	White-beaked dolphin (WBD)
**PCBs**											
Mean (±std. error)	11.9 ± 2.39	75.9 ± 14.7	18.6 ± 9.88	8.17 ± 2	16.3 ± 0.73	264 ± 82.52	8.46 ± 1.52	31.3 ± 2.95	6.93 ± 1.09	37.19 ± 11.96	26.90 ± 6.61
Max	54.9	698	158.5	34.2	159.7	956	31.3	225	12.0	183.6	124.4
Min	1.59	0.82	1.20	0.64	0.46	11.8	0.36	0.46	4.41	1.87	5.19
Relative abundance^b^.	52.4	70.1	81.1	82.7	71.0	52.1	71.9	85.1	47.8	55.1	64.3
% above threshold	0	80	31	17	42	100	31	70	17	13	85
**DDTs**											
Mean	11.0 ± 2.29	25.47 ± 8.94	1.61 ± 0.52	1.38 ± 0.64	3.45 ± 0.15	297 ± 118	2.24 ± 0.85	5.45 ± 0.72	7.81 ± 2.11	17.69 ± 8.3	12.3 ± 3.32
Max	31.0	219.0	4.65	4.67	42.7	1200	15.6	33.5	17.7	99.3	51.13
Min	0.89	0.50	0.32	0.16	0.00	27.4	0.07	0.09	3.53	1.5	2.51
Relative abundance	41.9	22.0	16.2	14.5	18.2	43.1	17.7	12.1	48.9	40.1	25.1
% above threshold	66.7	80	23	17	38	100	39	74	100	88	100
**PBDEs**											
Mean	0.38 ± 0.05	3.01 ± 0.74	0.18 ± 0.09	0.08 ± 0.02	0.90 ± 0.06	8.41 ± 3.63	0.58 ± 0.18	0.59 ± 0.15	ND	0.51 ± 0.17	3.06 ± 0.93
Max	0.61	15.4	0.80	0.14	15.7	25.5	2.53	1.46	ND	1.94	12.6
Min	0.09	0.08	0.03	0.03	0.02	0.69	0.03	0.03	ND	0.07	0.45
Relative abundance	2.13	4.95	1.46	1.30	6.77	1.31	5.24	1.20	ND	1.85	7.15
% above threshold	0	21	0	0	0	100	0	0	ND	0	44
**HCHs**											
Mean	0.11 ± 0.07	0.05 ± 0	0.03 ± 0	0.03 ± 0	0.10 ± 0.01	0.17 ± 0.07	0.04 ± 0.01	0.13 ± 0.02	0.02 ± 0	0.07 ± 0.03	0.07 ± 0.02
Max	0.90	0.12	0.05	0.04	2.02	0.58	0.10	0.90	0.02	0.37	0.27
Min	0.02	0.00	0.01	0.03	0.00	0.00	0.00	0.00	0.01	0.02	0.03
Relative abundance	0.22	0.21	0.61	0.78	0.50	0.06	0.91	0.70	0.15	0.34	0.20
**HCB**											
Mean	0.37 ± 0.05	0.39 ± 0.04	0.01 ± 0	0.01 ± 0	0.24 ± 0.01	2.24 ± 0.58	0.25 ± 0.05	0.17 ± 0.01	0.40 ± 0.08	0.30 ± 0.04	0.47 ± 0.04
Max	1.19	1.44	0.03	0.05	1.88	8.63	1.08	0.81	0.63	0.98	0.85
Min	0.20	0.01	0.01	0.01	0.00	0.33	0.02	0.00	0.09	0.15	0.21
Relative abundance	2.14	1.40	0.21	0.21	1.93	0.71	3.46	0.59	2.70	1.95	2.23
**Dieldrin**											
Mean	0.89 ± 0.61	0.66 ± 0.17	0.03 ± 0.01	0.04 ± 0.01	0.78 ± 0.06	20.6 ± 10.3	0.22 ± 0.09	0.48 ± 0.11	0.06 ± 0.01	0.55 ± 0.27	0.89 ± 0.44
Max	7.50	3.94	0.08	0.12	13.4	88.0	1.09	6.71	0.11	2.82	5.14
Min	0.03	0.02	0.01	0.01	0.00	0.11	0.01	0.01	0.03	0.04	0.05
Relative abundance	1.22	1.41	0.38	0.52	1.59	2.77	0.87	0.34	0.43	0.71	0.99
**Summedpollutants**^c^											
Mean	27.2 ± 6.87	95.2 ± 24.8	24.2 ± 17.4	7.82 ± 2.61	19.8 ± 0.84	657 ± 229	11.3 ± 2.63	37.9 ± 4.48	15.2 ± 3.25	56.9 ± 26.5	48.3 ± 13.5
Max	95.5	583	162	20.54	159.2	2000	47.3	244	30.4	286	180
Min	2.73	3.03	1.58	1.99	0.60	52.7	0.48	0.62	8.89	3.61	9.65

a Further details including sample size, congeners/isomers analyzed, pollutant applications, and year ratified in the Stockholm Convention are included in Tables S1 and Table S2. *Thresholds: PCBs 9 mg/kg lipid;^17,38^ pp-DDE 1.4 3 mg/kg lipid;^48^ PBDEs 1.5 mg/kg lipid.^47^ Concentrations are three significant figures.

b The concentration of each pollutant relative to the summed pollutant concentration.

c As per the spatiotemporal models, PBDEs were excluded from the summed pollutants calculations. The mean values here differ from the sum of the means above because of the different sample sizes. Only individuals with complete records for each pollutant were included in these summary figures. Please see Table S2 for details of the sample sizes for each pollutant.

Relative abundances of pollutants and congeners were highly variable between species, however, the three most abundant pollutants followed the same trend PCBs > DDTs (pp′DDE> pp′DDT) > PBDEs in all but one of the species we examined (sperm whales differed from the other species as the relative abundance of DDTs was higher than PCBs) (Table 2, Figure S2). The remaining pollutants (dieldrin, HCHs, and HCB) each contributed less than 3% to the overall concentration. We found higher ratios of ∑DDTs to ∑PCBs in longer lived species (Atlantic white-sided dolphins, killer whales, and striped dolphins) (Table S2). The contribution of PCBs to overall concentration was highest in gray seals (84%), and lowest in sperm whales (48%) (Figure S2). In all of the species we investigated, CB153 was the most abundant PCB compound. The median contribution of lower (*tri-, tetra-, and penta-*) chlorinated PCBs was significantly lower in pinnipeds compared with odontocetes (Kruskal–Wallis, *X*^2^(1, *N* = 1128) = 159, *p* < 2.2e^–16^, Dunn test, *z* = 12.6, *p* = 0) (Figure S2). BDE 47 was the most abundant PBDE congener, and the profiles, across all species, were dominated by lower brominated PBDEs (Figure S2).

### 3.2 Spatiotemporal Trends

**Figure 3:**
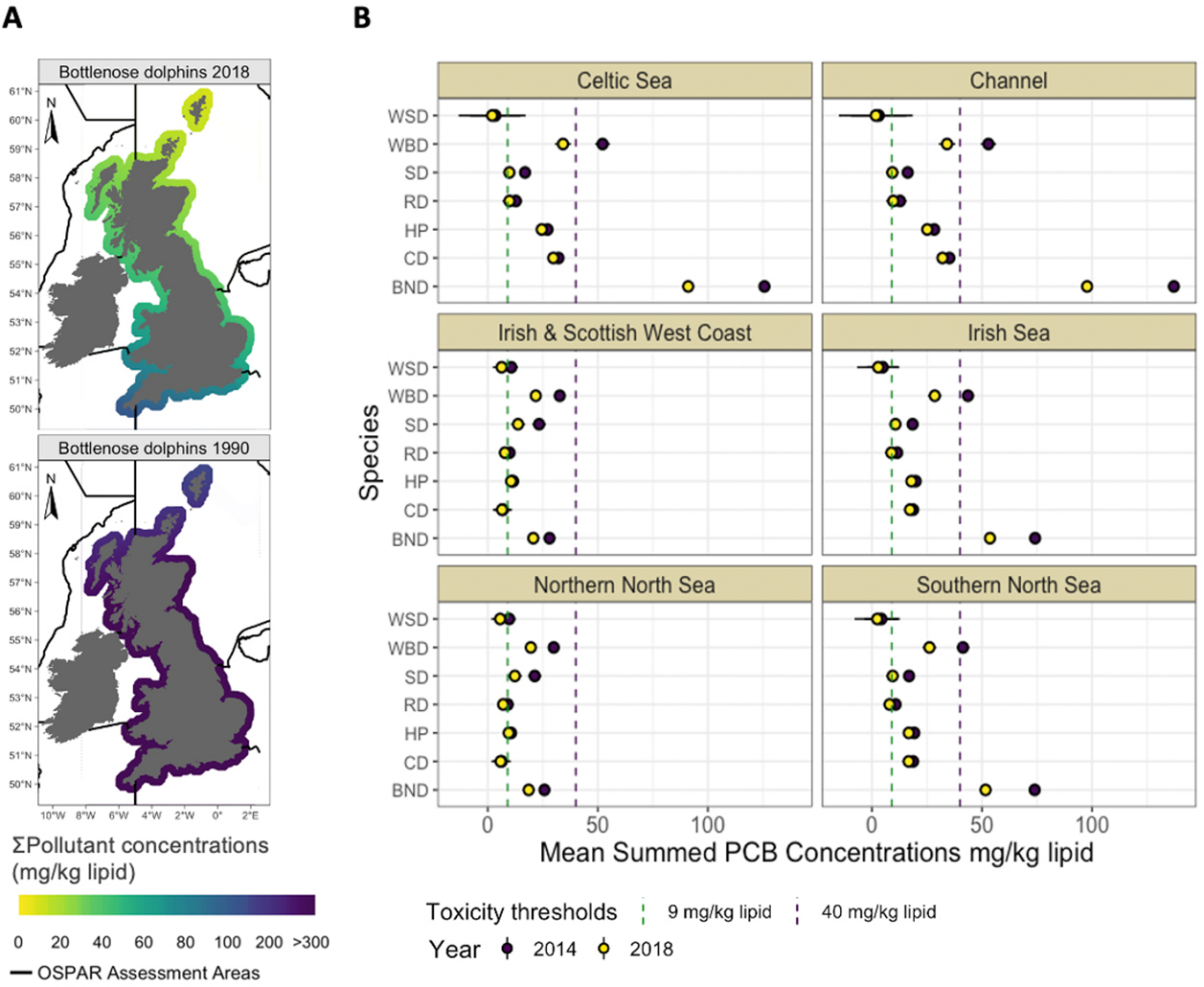
(A) Modeled spatial distribution of summed pollutant concentrations along the coast of Great Britain. Estimates were derived from the model for adult male bottlenose dolphins (BND) in 1990 and 2018. The black lines indicate the OSPAR contaminants assessment areas. Model coefficients are provided in Table S4. (B) Modeled mean PCB concentrations in adult males for each species in each OSPAR contaminants assessment area in 2014 and 2018. The abbreviations for each species are listed in Table 1. The horizontal bars represent twice the standard error. *Samples were not collected from the Irish coast; this area has been included for reference and to aid comparison with OSPAR contaminant assessments in other environmental compartments.

Our analysis of pollutant concentrations over time shows that concentrations of pollutants in marine mammals, that stranded along Great Britain, have declined over the last three decades, across all species (GLM, *p* < 0.05). However, there was considerable variation in pollutant concentrations and rates of decline among species and geographical regions (Figures 2, 3A and B, Figure S1, Table S3). Of all the pollutants analyzed, PCBs are declining at the slowest rate (β = −0.59, *p* = <. 05) and present in the highest concentrations across all species (Table 2, Figure S1, Table S4–S8).

Bottlenose dolphins had the highest modeled concentrations across all pollutant classes (GLM, *p* < 0.05) (Figure 2, Figure S1, Tables S3–S8), with the exception of HCB. However, it is important to note that killer whales had a far higher mean concentration of POPs than all other species (Table 2) but their low sample size and high variance meant they were excluded from the spatiotemporal analyses to preserve statistical robustness.

All species show the same pattern of pollutant concentration for age class and sex: adult males > juveniles > adult females. Modeled rates of pollutant declines were significantly slower in harbor porpoises and common dolphins (GLM, *p* < 0.05) than in other species, across all pollutants, except for HCH (Figure 2, Figure S1 and Tables S3–S8). Summed pollutant concentrations were highest at lower latitudes and rates of decline vary longitudinally such that levels are declining faster on the North Sea coast of Great Britain compared to the Atlantic coast (GLM, *p* < 0.05) (Figure 3A, Figure S1, Table S3). Bottlenose dolphins within the English Channel, the Celtic Sea, the Irish Sea, and Southern North Sea OSPAR (Oslo and Paris Conventions) assessment areas face a substantial threat as modeled mean concentrations exceeded the highest known threshold for toxic effects induced by PCBs in marine mammals (41 mg/kg lipid)^45^ (Figure 3B). Of the pollutant classes modeled, PCBs are declining slowest and have the greatest latitudinal concentration gradient, with concentrations decreasing from north to south (GLM, *p* < 0.05) (Figure S1, Table S4). In contrast, DDTs showed faster declines, almost twice that of PCBs, and exhibited a longitudinal gradient, whereby concentrations were higher on the west coast of Great Britain (GLM, *p* < 0.05) (Figure S1, Table S5). With the exception of PCBs, the spatial distributions of all pollutants have shifted longitudinally over time across all species (GLM, *p* < 0.05) (Figure S1, Tables S4–S8). In 1990, concentrations were highest in eastern regions, while in the more recent years of the study, concentrations were highest at western longitudes.

### 3.3 Risk Assessment of Pollutants

Comparing the tissue concentrations of pollutants against published toxicity thresholds, we found that marine mammals are still exposed to pollutants at levels that present a substantial toxicological risk to health. Of the individuals sampled within the most recent five years of the study (2014–2018), PCB concentrations in 48% (88/184) of individuals, exceeded the threshold for marine mammals for the onset of various physiological effects in marine mammals (9 mg/kg lipid)^17,38^ and 64% (118/184) exceeded the adverse effect concentration for lymphocyte proliferation (5.42 mg/kg lipid)^42^ (Table 1, Figure S3B, Table S1). Mean concentrations of PBDEs exceeded the only published threshold for toxic effects (1.5 mg/kg lipid)^47^ in killer whales, bottlenose dolphins and white-beaked dolphins (Table 2, Table S1) and in 8% of all individuals in the most recent five years of the study (Figure S3D). The distribution of concentrations in relation to these thresholds is shown in Figure S3. Over the same time period, concentrations of *p,p*′-DDE were above the minimum *p,p*′-DDE immunotoxic effect concentration reported for bottlenose dolphins in 55% of individuals (Table 2, Figure S3F).^48,56^ Despite the proportion of individuals exposed to toxic levels of pollutants decreasing over time, tissue concentrations in a substantial proportion of marine mammals remain at levels associated with impacts on health (Figure S3).

**Figure 4:**
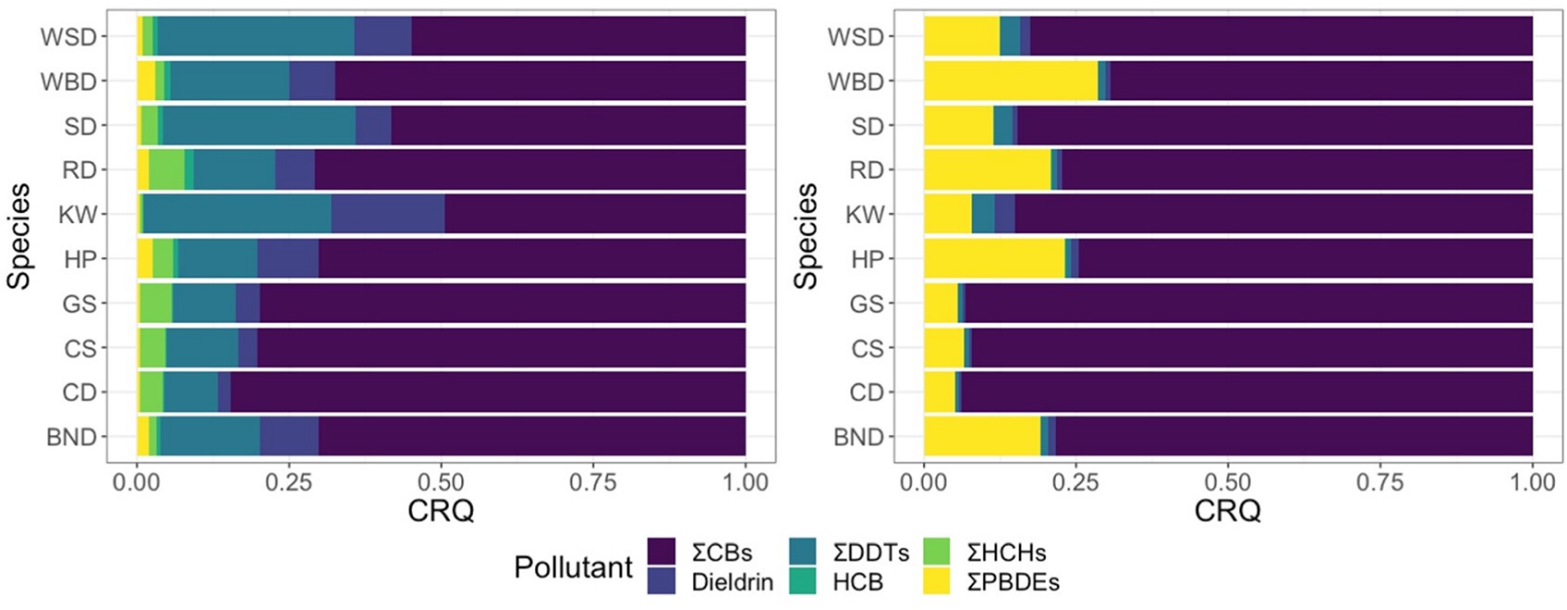
Relative contributions of each pollutant class to overall toxicity for each species for (A) endocrine disruption and (B) immunotoxicity. Relative contributions of each pollutant were calculated by dividing the CRQ for each pollutant by the summed CRQs. CRQs were derived using toxicity reference values (TRVs) for murine models (Table S9). The CRQs are shown in Table S10.

The relative toxicity quotients derived for each species demonstrate that PCBs pose the greatest risk to marine mammal health across both of the immunotoxic and endocrine disrupting end points we investigated (Figure 4 A and B, Table S10). The contribution of PCBs toward immunotoxicity ranged from 69% in Atlantic white-beaked dolphins to 93% in gray seals, while their contribution toward endocrine disruption ranged from 49% in killer whales to 80% in gray seals. Although PCBs represent the greatest risk across both end points, we found that rankings, in terms of risk, for the other pollutants varied depending on the end point being investigated. Of the other pollutants, DDTs represented the next highest risk to endocrine disruption with values ranging from 9% in gray seals to 32% in white-sided dolphins (Figure 4B). However, DDTs posed a much smaller risk with respect to immunotoxicity, with the proportion of risk ranging from 1% in common dolphins to 5% in sperm whales (Figure 4B).

## 4 Discussion

Here we show that, despite restrictions and bans on several persistent organic pollutants (POPs) in Europe, almost four decades ago,^57^ and worldwide, over 20 years ago,^5^ many marine mammals remain exposed to a widespread, persistent and toxic chemical threat from POPs. Although pollutant concentrations are declining, POPs are still present at concentrations that are likely to significantly impact marine mammal health and there is considerable variation in concentrations and rates of decline across species, pollutant classes, and regions. By monitoring sentinel species to assess the impacts of past and current elimination and mitigation actions, we have demonstrated that further efforts are required to protect marine mammals in the region. It is vital that governments do not lose sight of this issue, amid the context of other global pressures such as, climate change, impacts from fisheries and plastic pollution, as these threats can interact with POPs and increase environmental concentrations.^58,59^

### 4.1 Spatiotemporal Variation

We have revealed that, despite the decline in mean total POP concentrations in marine mammals over the last three decades, concentrations in a substantial proportion of individuals are still above toxicity thresholds, particularly in species feeding at higher trophic levels that stranded along more industrialized coastlines. This finding is consistent with other studies that have found higher levels of contaminants are positively correlated with trophic level.^60,61^ Pollutant concentrations are dependent on a number of factors (e.g., bioavailability, habitat, food web structure and feeding ecology^61−63^), which can affect spatial patterns. However, the spatial variation we observed could also be explained by geographical differences in historical use, contemporary discharges, and atmospheric transport dynamics indicating that these are also likely to be primary drivers of spatial patterns. Levels of industrialization are greater at lower latitudes in Great Britain e.g., PCBs were manufactured at a single site on the Bristol channel.^64^ As such, legacy and contemporary releases are more likely to have occurred at lower latitudes, as reflected by the spatial distribution in PCB concentrations and congener relative abundances. Spatial patterns of the other pollutants, which were primarily used as crop treatments, also appear to reflect their historical use. Concentrations are higher and have declined at a slower rate in the North Sea, which is associated with greater arable farming effort.^65^

Our analysis also revealed temporal shifts in the spatial distribution of pollutants. Dispersal of legacy pollutants over time has been well documented and is thought to be driven either by concentration gradients or by latitudinal temperature gradients, from warmer to cooler areas.^66,67^ Our findings suggest that the dominant dispersal mechanism may vary across pollutant classes. Dispersal of PCBs over time showed a greater latitudinal gradient (which may be driven by environmental variables (e.g., atmospheric currents or temperature) and concentration gradients) while the dispersal of other pollutants appeared to correlate with longitude, which is more likely to be primarily driven by concentration gradients (Figure S1). This is demonstrated by the shift in distribution of the pesticides DDT, HCB, dieldrin, and HCH, with higher concentrations found on the North Sea compared to the Atlantic coast earlier in the study and vice versa in more recent years (Figure S1, Tables S5–S8). This evidence of atmospheric transport is also concerning as atmospheric long-range transport from secondary and primary sources is the major input of PCBs in pristine environments such as the Arctic.^68^ Recent research has, however, demonstrated that air concentrations of most POPs are generally decreasing or not increasing in the Arctic, with the exception of HCB.^68^

It is important to consider that the spatial distribution of pollutant concentrations can also vary according to animal movement and carcass drift, causing animals to accrue pollutants in a location different from where they strand. It is likely that several individuals will have foraged in waters at large distances from their stranding location and so pollutant levels are likely to be reflective of the wider regions.^69^ We expect our results are representative of marine mammals that inhabit the seas surrounding Great Britain; however, there may be local variations. For example, there are large numbers of common dolphins in the southwest of the UK; however, they are a wide ranging and fast-moving species and little is known about the movement of individual pods; therefore, spatial trends should be interpreted with caution. Similarly, killer whales in the UK are most commonly found in Scottish waters but have been spotted as far south as Cornwall with relatively short time periods between sightings. While it was not possible to determine an animal’s movements and foraging activity over their lifespan, we were able to minimize the impact of carcass drift by prioritizing samples that were fresh or only slightly decomposed (933/1070 = 83%). It is also important to note that older or younger “weaker” individuals may be overrepresented in stranding data, which could confound our results. We attempted to minimize the influence of these biases by controlling for age class and conditions in our models. We were unable to subset the data to only include trauma cases, as this would have excluded five of the seven species from the analyses. However, a high proportion of trauma cases, which are considered to represent the source population exposure, were included in the analysis (*n* = 377).^70^

### 4.2 Inter- and Intraspecific Differences

Inter- and intraspecific differences such as lifespan, metabolic capabilities, foraging strategies and trophic level can cause some species and individuals to be at greater risk of high pollutant concentrations than others.^71^ Concentrations tend to be greater in longer lived species that feed at a high trophic level, as evidenced by the high concentrations that we found in killer whales and bottlenose dolphins. We also observed high levels of variation among individual killer whales, which is likely to be the result of differences in feeding ecology. Sympatric killer whale populations in Norway and Iceland have been shown to have vastly different levels of pollutants due to interindividual variation in prey specialization.^60,72^ This is likely to be the case in killer whales in UK waters as the resident population is known to be marine mammal eating while transient killer whales feed primarily on fish however, recent evidence has shown some individuals switch between fish and marine mammal prey.^73^ Concentrations were higher in juveniles than adult females, which is reflective of lactational transfer of lipophilic pollutant burdens from mothers to calves and has been associated with reduced calf survival.^74^ Differences in pollutant concentrations between species can also occur if the home ranges of some species are more contaminated than others.^75^ We found concentrations were highest in animals that stranded at low latitudes along the west coast of Great Britain, an area associated with higher levels of industrialization and the production of PCBs. This area is inhabited by a large number of common dolphins, which may explain why concentrations in this species are falling at a significantly slower rate than other species.^76^ It is important to note that interspecific comparisons of pollutant concentrations may be confounded by differences in the blubber thickness. We attempted to control for this by including the blubber thickness in our models. Species comparisons may also be confounded by an over presentation of older individuals. We were only able to control for age class and sex as yearly age was unavailable for around half of the individuals (*n* = 512). Nevertheless, among the individuals with available age data, we observed an even distribution of the adult ages. In addition, our interspecies comparisons were consistent with other studies.^17^

By analyzing a broad spectrum of pollutants, we were able to use relative abundances of pollutants to infer spatial differences in contamination sources as well as intraspecific differences in metabolism and lifespan. Concentrations in long-lived species can represent multidecadal exposure and hence relative abundances in these species often lag behind those found in the environment and shorter-lived biota.^77^ The higher ∑DDTs to ∑PCBs ratios we found in longer-lived species (Atlantic white-sided dolphins, killer whales, and striped dolphins) provide evidence of this and reflect the faster rate of decline in ∑DDTs concentrations, which may be due to the greater persistence of PCBs or continued environmental contamination. We found that BDE47 was the most abundant PBDE congener, which is reflective of the likely contamination source, as BDE47 is associated with the legacy production of the penta-BDE commercial mixture. It is estimated large reservoirs of this compound are still in circulation.^78^ Ratios of pollutants can also vary according to the metabolic capabilities between species. For example, pinnipeds are able to metabolize some pollutants (lower chlorinated PCB congeners and all PBDEs) more easily than odontocetes^79^ and have vastly shorter lactation periods,^80^ as demonstrated by the differences in PCB homologues we observed. Pollutant concentrations and abundance profiles can also be affected by loss of blubber mass, as a consequence of chronic negative energy balance.^6,9,81^ We were able to minimize this by controlling for variation in blubber thickness.

### 4.3 Risk Assessment

We have shown that marine mammals are exposed to a barrage of legacy pollutants and it is now well documented that antagonistic and synergistic actions of pollutants can create toxic mixtures, even when each pollutant is present at a level deemed to be safe.^82^ Therefore, toxic thresholds can be considered to be conservative, as the toxicological risk that marine mammals face is likely to be exacerbated by the effects of the mixture of pollutants to which they are exposed. We identified exposure to PCBs as the greatest risk to health, in terms of their contribution to toxicity and slow decline in comparison to other pollutants. Other pollutants are present at toxic concentrations and account for a substantial proportion of the risk to the immune and endocrine systems. Accurate risk assessments of detrimental effects of POPs on marine mammals are possible but require an interdisciplinary response across ecology, ecotoxicology, and analytical chemistry and will require large amounts of resources to obtain accurate estimates for each species. Given the lack of toxicological information that is available for marine mammals, the use of Kannan et al.’s threshold is appropriate to assess population risk and allows for comparisons to be made across different studies. Moreover, the threshold was derived using results from toxicity studies on free ranging and captive marine mammal species.^48,83−86^

To compare the relative toxicity of the pollutants, we have used values derived for murine models, in lieu of marine mammal toxicity reference values. We note there are likely to be considerable levels of uncertainty and that there are anatomical and physiological differences between species that will affect the absorption, distribution, metabolism, and excretion of pollutants and toxicities. However, our goal was not to define absolute toxicity but to compare relative toxicities. We think, therefore, in the absence of marine mammal data and accurate pharmacokinetic models this approach can provide meaningful insights and is routinely used in ecotoxicology.^87^ It is clear that exposure to multiple pollutants is likely to increase the risk of harmful effects and should be accounted for when determining acceptable environmental concentrations and managing contamination.^82^ Even without taking mixture effects into consideration, we demonstrated that a substantial number of individuals are exposed to single pollutants at levels deemed to be a toxicological risk. Animals that have pollutant concentrations that exceed toxic thresholds are likely to have a reduced ability to fight infectious disease and reproduce successfully;^88^ therefore, current pollutant exposures may be causing population level impacts.

Aside from exposures to pollutants, marine mammals face an increasing number of threats, and population level impacts are likely to be exacerbated in areas where high levels of contamination coincide with other pressures (e.g., acoustic disturbance, prey depletion, and climate change). For example, we have shown that pollutant concentrations in common dolphins are a persistent threat and are declining at a rate significantly slower than those of other species. When combined with the substantial threat they face from by-catch and their conservation status, as defined in the latest assessment for the EU Habitats Directive, of “unknown” in the UK and Atlantic,^89,90^ our findings raise concerns about the long-term health of this population. The impact of pollutant exposure may also be greater in areas where high levels of contamination overlap with those of biological importance. We have shown that Cardigan Bay, one of a network of protected sites set out by the European Union’s Habitat Directive known as Special Areas for Conservation (SACs), is located in an area associated with higher pollutant exposures. Further, the SAC is in close proximity to a major UK PCB manufacturing site at Newport that dumped 800 000 tons of waste contaminated with PCBs into a porous lime-stone quarry and so may be more vulnerable to contemporary releases.^64^ Remediation work has been carried out however, there is ongoing controversy as to whether waste was dumped into other quarries in the area.^91^ We have demonstrated that a harmonized and integrated approach is needed to monitor and assess marine mammal health in relation to pollution and other combined pressures, particularly in areas of high ecological value such as Marine Protected Areas and SACs.

Our findings have highlighted the importance of examining pollutant concentration heterogeneity within populations alongside overall spatiotemporal trends to assess whether mitigation actions are sufficient to protect vulnerable wildlife and ecosystems. We have shown that despite overall concentrations of POPs declining, they remain a threat in several species and regions. Given the pervasive and persistent threat of chemical contamination in marine mammals, our findings are globally significant and highlight the need to ensure that environmental concentrations of persistent organic pollutants continue to decline.

## Data Availability

Aggregated data that support the findings of this study are available to download as part of the Supporting Information. Some of the raw data (e.g., stranding location) cannot be made public due to challenges in ascribing ownership to multiple contributors who were funded through various grants, leading to complexities in the publication of the unprocessed data. However, all of the raw data are available from the authors on request.

## Supporting Information Available

Aggregated data (XLSX)
Model coefficients for each pollutant class (Figure S1); relative abundance plots (Figure S2); relative frequencies of concentrations (Figure S3); additional details on congeners and isomers that were analyzed (Table S1); sample size, mean and max concentrations of pollutants (Table S2); model coefficients for summed concentrations of pollutants (Table S3); model coefficients for PCBs (Table S4); model coefficients for DDTs (Table S5); model coefficients for HCB (Table S6); model coefficients for dieldrin (Table S7); model coefficients for HCHs (Table S8); toxicity reference values (Table S9); comparative risk quotients (Table S10); sample sizes for unpublished and previously published data (Table S11); pollutant percentage detections (Table S12) (PDF)
