# Supplementary material for: Spatiotemporal Trends Spanning Three Decades Show Toxic Levels of Chemical Contaminants in Marine Mammals
Source: Environ Sci Technol. 2023 Nov 27;57(49):20736–49. doi: 10.1021/acs.est.3c01881 (PMC10720377; doi:10.1021/acs.est.3c01881)
Supplement: Supplementary file 2 — es3c01881_si_002.pdf [file es3c01881_si_002.pdf]

Supplementary Information for the manuscript titled: "Spatiotemporal trends spanning three decades show toxic levels of chemical contaminants in marine mammals"

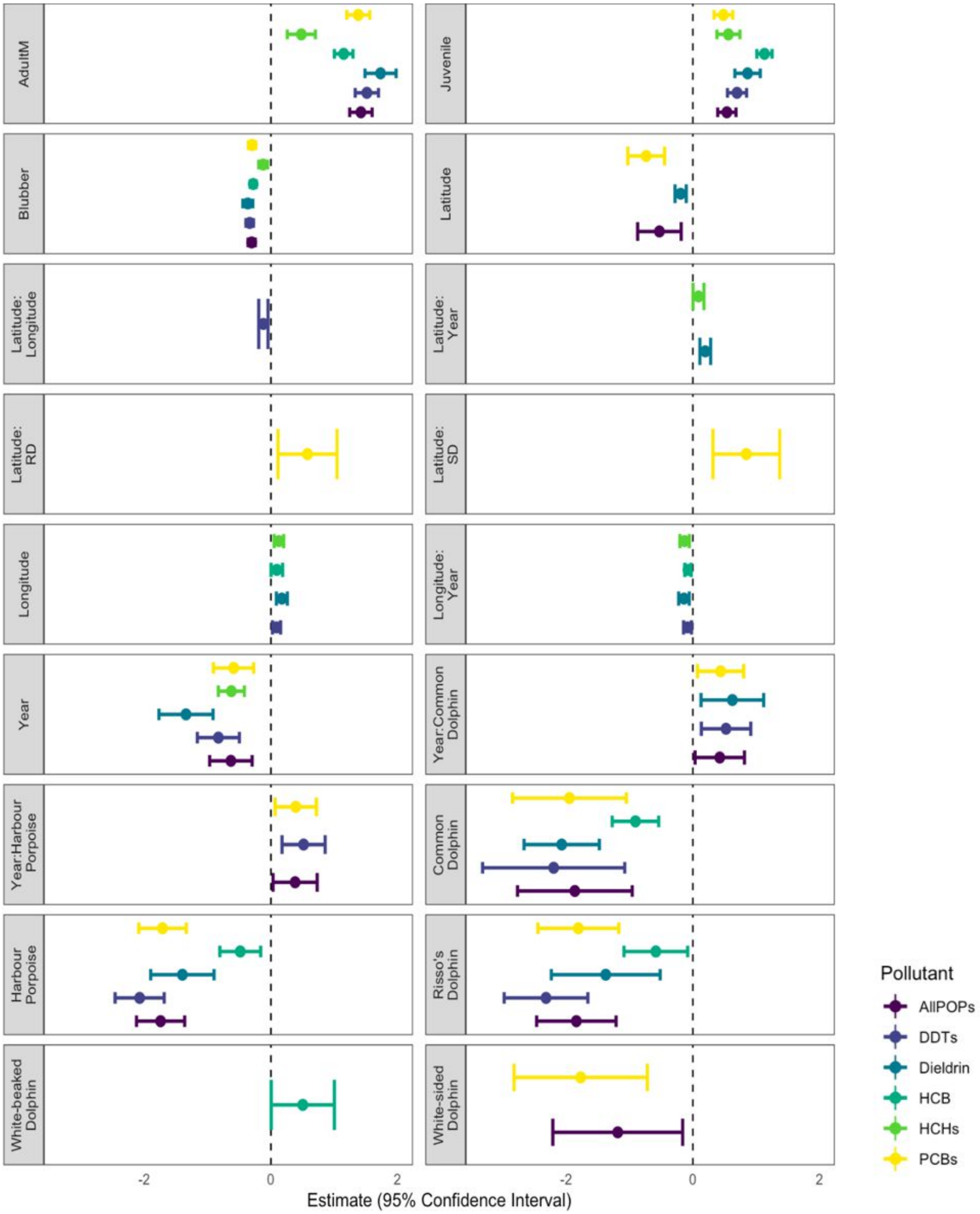

Figure S1: Forest plot of the averaged model coefficients for each pollutant class.
The dots represent the coefficient estimates from the models, the upper and lower limits represent the 95% confidence intervals. Only significant terms have been included (GLM, p<0.05).(SD (Striped Dolphin), RD (Risso's Dolphin).

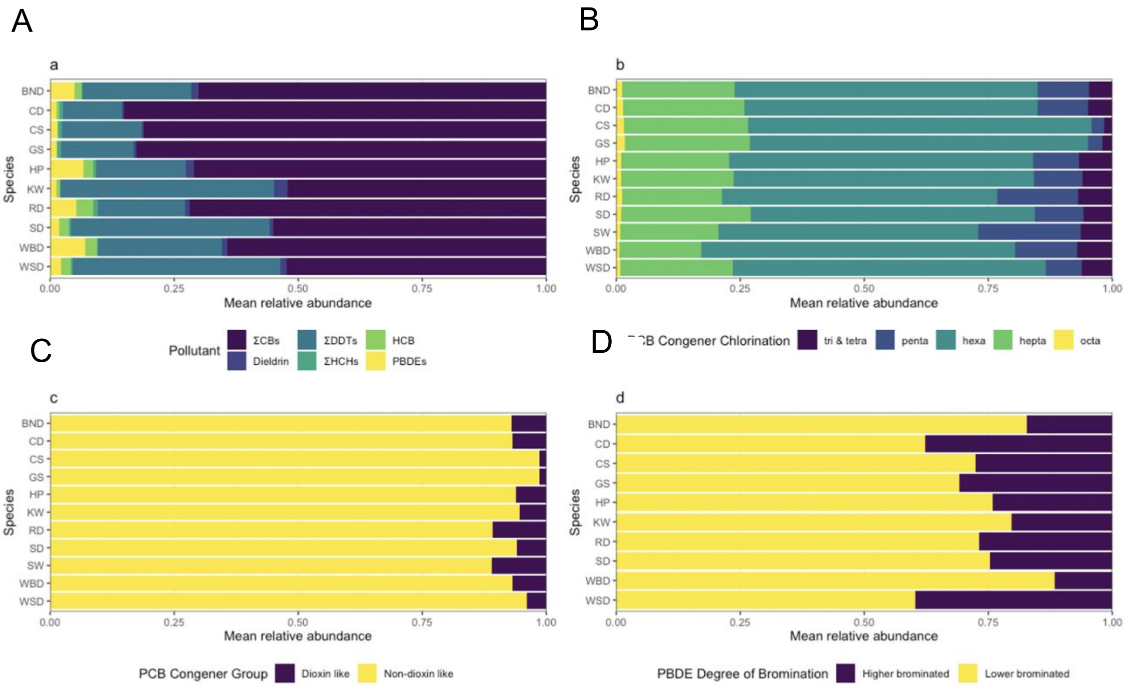

BND	CD	CS	GS	HP	KW	RD	SD	SW	WBD	WSD
Bottlenose dolphin	Short-beaked common dolphin	Common seal	Grey seal	Harbour porpoise	Killer whale	Risso's dolphin	Striped dolphin	Sperm whale	White-beaked dolphin	Atlantic white-sided dolphin

Figure S2: Mean relative abundance plots for each species.
(A)Pollutant class (B)Polychlorinated biphenyls congeners grouped by chlorination group (PCB homologs) (C)Polychlorinated biphenyls congeners grouped by dioxin (CBs 105, 118, 156) or non-dioxin group (CBs 18, 28, 31, 44, 47, 49, 52, 66, 101, 110, 128, 138, 141, 149, 151, 153, 158, 170, 180, 183, 187, 194) (D) Polybrominated diphenyl ethers congeners grouped by degree of bromination, higher brominated (BDEs 138, 153, 154, 183), lower brominated (BDEs 17, 28, 47)

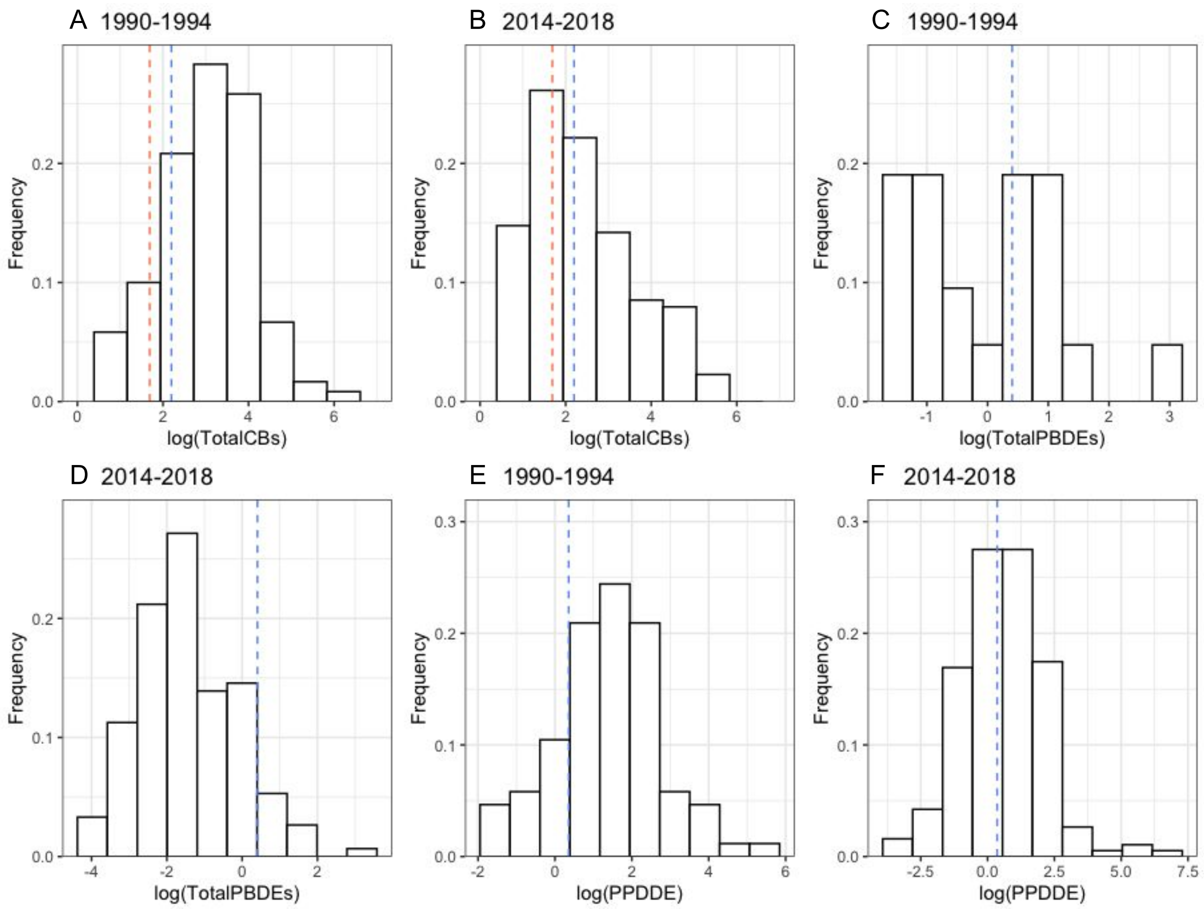

Figure S3: Relative frequencies of pollutant concentrations again selected published toxic thresholds in marine mammals.

The data have been split into time periods covering the first five years of the study period (1990-1994) and the most recent five years of the study period (2014-2018). PCBs (red line: Desforges et al., 2016[2]; blue line: Kannan et al., 2000[1]), PBDEs (blue line: Hall et al., 2003[3]), pp'-DDE (blue line: Lahvis et al., 1995[4]). *Toxicological endpoints for each threshold are shown in Table 1 of the manuscript.*

Table S1: The congeners, isomers and historical applications of each class of persistent organic pollutant (POPs) analysed in the blubber.

**Pollutant Class**	**Congeners/isomers analysed**	**Applications**	**Year added to Stockholm Convention**	**Reference**
Polychlorinated biphenyls (PCBs) Dioxin-like congeners are marked with an asterisk (*)	CB18, CB28, CB31, CB44, CB 47, CB49, CB52, CB66, CB101, CB105*, CB110, CB118*, CB128, CB138, CB141, CB149, CB151, CB153, CB156*, CB158, CB170, CB180, CB183, CB187, CB194	Used in construction joint sealants, paints, transformers and capacitors, lubricants, plasticizers	2001	(Stuart-Smith & Jepson, 2017) [5]
Dichlorodiphenyltrichloroethane (DDTs)	*p,p'*-DDE, *p,p'*-DDT, *p,p'*-TDE	Insecticide	2001	(Pacyna et al., 2003) [6]
Polybrominated diphenyl ethers (PBDEs)	BDE17, BDE28, BDE47, BDE66, BDE85, BDE99, BDE100, BDE138, BDE153, BDE154, BDE183	Flame retardants used in electrical equipment, construction materials, textiles, furniture foam, plastics	2009	(Evenset et al., 2007) [7]
Hexachlorocyclohexanes (HCHs)	α-HCH, γ-HCH	Pesticide	2009	(Pacyna et al., 2003) [6]
Hexachlorobenzene (HCB)	HCB	Fungicide, combustion by-product	2001	[6] (Pacyna et al., 2003) [6]
Dieldrin	Dieldrin	Insecticide	2001	(Honeycutt & Shirley, 2014) [8]

Table S2: Sample size, mean and maximum concentrations (mg/kg lipid) of each persistent organic pollutant in each species.

	Pollutant class and lipid content								
	LIPID	PCBs	DDTs	DDT/DDE	DDT/PCB	Dieldrin	PBDEs	HCHs	HCB
Species	N(sample size) *bold number indicates sample size included in models								
Atlantic white-sided dolphin	23	22 **(11)**	12 **(11)**	12 **(11)**	12	12 **(11)**	11	12 **(11)**	22 **(11)**
Bottlenose dolphin	63	63 **(28)**	33 **(28)**	33 **(28)**	33	33 **(28)**	28	33 **(28)**	63 **(28)**
Common seal	16	16	9	9	9	9	9	9	16
Grey seal	21	21	7	7	7	7	7	7	21
Harbour porpoise	784	731 **(604)**	658 **(604)**	657 **(604)**	653	658 **(604)**	420	658 **(604)**	736 **(604)**
Killer whale	15	15	10	10	10	10	8	10	15
Risso's dolphin	26	26 **(14)**	18 **(14)**	18 **(14)**	18	18 **(14)**	15	18 **(14)**	26 **(14)**
Short-beaked common dolphin	125	124 **(63)**	80 **(63)**	80 **(63)**	80<br>6	80 **(63)**	10	80 **(63)**	125 **(63)**
Sperm whale	6	6	6	6	12	6	0	6	6
Striped dolphin	24	22 **(11)**	12 **(11)**	12 **(11)**	15	12 **(11)**	11	12 **(11)**	23 **(11)**
White-beaked dolphin	25	24 **(14)**	15 **(14)**	15 **(14)**	12	15 **(14)**	14	15 **(14)**	24 **(14)**
	Mean blubber concentration (mg/kg lipid)								
Atlantic white-	76.67	11.9	11.04	0.84	0.81	0.89	0.38	0.11	0.37

sided dolphin									
Bottlenose dolphin	56.25	75.85	25.47	0.81	0.35	0.66	3.01	0.05	0.39
Common seal	75.98	18.61	1.61	0.91	0.21	0.03	0.18	0.03	0.01
Grey seal	77.75	8.17	1.38	0.88	0.18	0.04	0.08	0.03	0.01
Harbour porpoise	83.56	16.31	3.45	0.62	0.27	0.78	0.9	0.1	0.24
Killer whale	51.05	263.81	297.38	0.91	0.98	20.62	8.41	0.17	2.24
Risso’s dolphin	63.34	8.46	2.24	0.79	0.25	0.22	0.58	0.04	0.25
Short-beaked common dolphin	77.13	31.27	5.45	0.68	0.16	0.48	0.59	0.13	0.17
Sperm whale	51.42	6.93	7.81	0.82	1.05	0.06	NA	0.02	0.4
Striped dolphin	71.37	37.19	17.69	0.84	0.72	0.55	0.51	0.07	0.3
White-beaked dolphin	76.56	26.9	12.26	0.86	0.4	0.89	3.06	0.07	0.47
					Maximum blubber concentration (mg/kg lipid)				
Atlantic white-sided dolphin	91.33	54.87	31.03	0.93	1.45	7.5	0.61	0.9	1.19
Bottlenose dolphin	89.6	697.99	219.02	0.97	1.43	3.94	15.37	0.12	1.44

Common seal	91.05	158.45	4.65	0.93	0.37	0.08	0.8	0.05	0.03
Grey seal	97.4	34.22	4.67	0.95	0.3	0.12	0.14	0.04	0.05
Harbour porpoise	100	159.68	42.78	0.97	1.84	13.41	15.65	2.02	1.88
Killer whale	73.72	956.37	1203.41	0.98	1.55	88.03	25.53	0.58	8.63
Risso's dolphin	88	31.3	15.61	0.9	0.72	1.09	2.53	0.1	1.08
Short-beaked common dolphin	92	225.1	33.49	0.92	0.47	6.71	1.46	0.9	0.81
Sperm whale	59.6	11.98	17.74	0.86	1.48	0.11	NA	0.02	0.63
Striped dolphin	92.41	183.61	99.28	0.96	1.22	2.82	1.94	0.37	0.98
White-beaked dolphin	93.24	124.35	51.13	0.96	0.5	5.14	12.63	0.27	0.85

Table S3: Model averaged coefficients for the variables that were modelled against the summed blubber concentrations of all pollutants.

	Estimate	Std. Error	Adjusted SE	Z value	Pr(>|z|)
(Intercept)	3.67	0.20	0.20	18.50	0.00
Age Group & Sex: adultM	1.42	0.09	0.09	15.75	0.00
Age Group: Juvenile	0.54	0.07	0.07	7.32	0.00
Blubber thickness	-0.30	0.03	0.03	10.53	0.00
Latitude	-0.53	0.18	0.18	3.01	0.00
Longitude	0.01	0.03	0.03	0.37	0.71
Days since 01/01/1990	-0.63	0.17	0.17	3.70	0.00
Common dolphin	-1.86	0.46	0.46	4.03	0.00
Harbour porpoise	-1.74	0.19	0.19	8.98	0.00
Risso’s dolphin	-1.84	0.32	0.32	5.75	0.00
Striped dolphin	-0.47	0.52	0.52	0.90	0.37
White-beaked dolphin	-0.13	0.35	0.35	0.36	0.72
White-sided dolphin	-1.19	0.52	0.52	2.27	0.02
Latitude:Longitude	-0.04	0.04	0.04	1.03	0.30
Latitude:Common dolphin	-0.06	0.29	0.29	0.22	0.82
Latitude:Harbour porpoise	0.18	0.18	0.18	1.01	0.31
Latitude:Risso’s dolphin	0.35	0.31	0.31	1.10	0.27
Latitude:Striped dolphin	0.48	0.41	0.41	1.17	0.24
Latitude:White-beaked dolphin	0.23	0.29	0.29	0.79	0.43
Latitude:White-sided dolphin	0.62	0.69	0.69	0.90	0.37
Longitude:days since 01/01/1990	-0.06	0.03	0.03	1.81	0.07
Days since 01/01/1990:Common dolphin	0.43	0.20	0.20	2.14	0.03
Days since 01/01/1990:Harbour porpoise	0.38	0.18	0.18	2.15	0.03
Days since 01/01/1990:Risso’s dolphin	0.17	0.25	0.25	0.68	0.50
Days since 01/01/1990:Striped dolphin	-0.29	0.35	0.35	0.82	0.41
Days since 01/01/1990:White-beaked dolphin	-0.06	0.27	0.27	0.24	0.81
Days since 01/01/1990:White-sided dolphin	-0.12	0.53	0.53	0.23	0.82
Latitude:days since 01/01/1990	0.01	0.02	0.02	0.33	0.74
Latitude:Longitude:Days since 01/01/1990	0.00	0.01	0.01	0.13	0.90

Table S4: Model averaged coefficients for selected variables against blubber concentrations of PCBs.

	Estimate	Std. Error	Adjusted SE	z value	Pr(>|z|)
(Intercept)	3.40	0.20	0.20	17.27	0.00
Age Group & Sex: AdultM	1.38	0.09	0.09	14.86	0.00
Age Group: Juvenile	0.48	0.08	0.08	6.41	0.00
Blubber thickness	-0.30	0.03	0.03	10.09	0.00
Latitude	-0.74	0.15	0.15	4.98	0.00
Days since 01/01/1990	-0.59	0.16	0.16	3.64	0.00
Common dolphin	-1.95	0.46	0.46	4.25	0.00
Harbour porpoise	-1.71	0.19	0.19	8.92	0.00
Risso’s dolphin	-1.81	0.32	0.33	5.55	0.00
Striped dolphin	-0.58	0.52	0.52	1.10	0.27
White-beaked dolphin	-0.13	0.36	0.36	0.36	0.72
White-sided dolphin	-1.77	0.54	0.54	3.30	0.00
Latitude:Common dolphin	-0.02	0.36	0.36	0.06	0.96
Latitude:Harbour porpoise	0.30	0.15	0.15	1.96	0.05
Latitude:Risso’s dolphin	0.58	0.24	0.24	2.43	0.02
Latitude:Striped dolphin	0.84	0.27	0.27	3.14	0.00
Latitude:White-beaked dolphin	0.48	0.31	0.31	1.56	0.12
Latitude:White-sided dolphin	1.23	0.67	0.67	1.84	0.07
Days since 01/01/1990:Common dolphin	0.44	0.19	0.19	2.36	0.02
Days since 01/01/1990:Harbour porpoise	0.39	0.17	0.17	2.35	0.02
Days since 01/01/1990:Risso’s dolphin	0.11	0.26	0.26	0.44	0.66
Days since 01/01/1990:Striped dolphin	-0.40	0.37	0.37	1.09	0.27
Days since 01/01/1990:White-beaked dolphin	-0.18	0.29	0.29	0.62	0.54
Days since 01/01/1990:White-sided dolphin	-0.40	0.58	0.58	0.69	0.49
Longitude	-0.01	0.03	0.03	0.26	0.79
Longitude:Days since 01/01/1990	-0.03	0.04	0.04	0.76	0.45
Latitude:Longitude	-0.02	0.03	0.03	0.55	0.58
Latitude:Days since 01/01/1990	0.01	0.02	0.02	0.38	0.70
Latitude:Longitude:Days since 01/01/1990	0.00	0.01	0.01	0.10	0.92

Table S5: Model averaged coefficients for selected variables against blubber concentrations of DDTs.

	Estimate	Std. Error	Adjusted SE	z value	Pr(>|z|)
(Intercept)	2.08	0.20	0.20	10.29	0.00
Age Group & Sex: AdultM	1.51	0.09	0.09	16.16	0.00
Age Group: Juvenile	0.70	0.08	0.08	9.06	0.00
Blubber thickness	-0.34	0.03	0.03	11.13	0.00
Latitude	-0.22	0.20	0.20	1.14	0.25
Longitude	0.09	0.03	0.03	2.73	0.01
Days since 01/01/1990	-0.83	0.17	0.17	4.90	0.00

Common dolphin	-2.20	0.57	0.57	3.84	0.00
Harbour porpoise	-2.07	0.20	0.20	10.48	0.00
Risso's dolphin	-2.32	0.34	0.34	6.87	0.00
Striped dolphin	0.23	0.52	0.52	0.44	0.66
White-beaked dolphin	-0.13	0.36	0.36	0.37	0.71
White-sided dolphin	-0.58	0.49	0.49	1.18	0.24
Latitude:Longitude	-0.12	0.04	0.04	3.18	0.00
Latitude:Common dolphin	-0.18	0.32	0.32	0.57	0.57
Latitude:Harbour porpoise	0.19	0.20	0.20	0.92	0.36
Latitude:Risso's dolphin	0.36	0.37	0.37	0.98	0.33
Latitude:Striped dolphin	0.25	0.30	0.30	0.83	0.41
Latitude:White-beaked dolphin	0.07	0.24	0.24	0.27	0.79
Latitude:White-sided dolphin	0.29	0.57	0.57	0.51	0.61
Longitude:Days since 01/01/1990	-0.08	0.03	0.03	2.63	0.01
Days since 01/01/1990:Common dolphin	0.52	0.20	0.20	2.64	0.01
Days since 01/01/1990:Harbour porpoise	0.52	0.17	0.17	2.97	0.00
Days since 01/01/1990:Risso's dolphin	0.29	0.26	0.26	1.12	0.26
Days since 01/01/1990:Striped dolphin	-0.10	0.37	0.38	0.28	0.78
Days since 01/01/1990:White-beaked dolphin	0.18	0.30	0.30	0.59	0.55
Days since 01/01/1990:White-sided dolphin	0.07	0.49	0.49	0.13	0.89
Latitude:Days since 01/01/1990	0.01	0.02	0.02	0.39	0.70
Latitude:Longitude:Days since 01/01/1990	0.00	0.02	0.02	0.27	0.79

Table S6: Model averaged coefficients for selected variables against blubber concentrations of HCB.

	Estimate	Std. Error	Adjusted SE	z value	Pr(>|z|)
(Intercept)	-2.12	0.17	0.17	12.54	0.00
Age Group & Sex: AdultM	1.15	0.07	0.07	15.38	0.00
Age Group: Juvenile	1.13	0.06	0.06	18.48	0.00
Blubber thickness	-0.28	0.02	0.02	11.66	0.00
Latitude	0.04	0.05	0.05	0.87	0.39
Longitude	0.09	0.05	0.05	2.01	0.04
Days since 01/01/1990	-0.17	0.15	0.15	1.13	0.26
Common dolphin	-0.91	0.19	0.19	4.83	0.00
Harbour porpoise	-0.48	0.16	0.16	2.94	0.00
Risso’s dolphin	-0.58	0.26	0.26	2.28	0.02
Striped dolphin	-0.19	0.34	0.34	0.55	0.58
White-beaked dolphin	0.50	0.25	0.25	1.98	0.05
White-sided dolphin	0.12	0.27	0.27	0.44	0.66
Latitude:Longitude	-0.02	0.03	0.03	0.64	0.52
Longitude:Days since 01/01/1990	-0.08	0.03	0.03	3.04	0.00
Days since 01/01/1990:Common dolphin	0.14	0.22	0.22	0.63	0.53
Days since 01/01/1990:Harbour porpoise	0.08	0.15	0.15	0.58	0.56
Days since 01/01/1990:Risso’s dolphin	0.11	0.20	0.20	0.55	0.58
Days since 01/01/1990:Striped dolphin	0.13	0.25	0.25	0.51	0.61
Days since 01/01/1990:White-beaked dolphin	0.08	0.17	0.17	0.46	0.65
Days since 01/01/1990:White-sided dolphin	0.09	0.19	0.19	0.45	0.65
Latitude:Days since 01/01/1990	0.00	0.02	0.02	0.28	0.78
Latitude:Longitude:Days since 01/01/1990	0.00	0.01	0.01	0.15	0.88
Longitude:Common dolphin	-0.01	0.09	0.09	0.16	0.88
Longitude:Harbour porpoise	-0.01	0.04	0.04	0.13	0.90
Longitude:Risso’s dolphin	0.00	0.04	0.04	0.07	0.95
Longitude:Striped dolphin	0.00	0.06	0.06	0.02	0.98
Longitude:White-beaked dolphin	0.00	0.05	0.05	0.09	0.93
Longitude:White-sided dolphin	0.00	0.04	0.04	0.00	1.00
Latitude:Common dolphin	-0.01	0.07	0.07	0.12	0.91
Latitude:Harbour porpoise	-0.01	0.04	0.04	0.14	0.89
Latitude:Risso’s dolphin	0.00	0.03	0.03	0.06	0.96
Latitude:Striped dolphin	-0.01	0.06	0.06	0.13	0.90
Latitude:White-beaked dolphin	-0.01	0.07	0.07	0.14	0.89
Latitude:White-sided dolphin	-0.01	0.05	0.05	0.09	0.92

Table S7: Model averaged coefficients for selected variables against blubber concentrations of dieldrin

	Estimate	Std. Error	Adjusted SE	z value	Pr(>|z|)
(Intercept)	-1.29	0.26	0.26	4.88	0.00
Age Group & Sex: AdultM	1.73	0.13	0.13	13.81	0.00
Age Group: Juvenile	0.86	0.10	0.10	8.45	0.00
Blubber thickness	-0.36	0.04	0.04	9.13	0.00
Latitude	-0.19	0.05	0.05	4.25	0.00
Longitude	0.17	0.04	0.04	3.92	0.00
Days since 01/01/1990	-1.34	0.22	0.22	6.15	0.00
Common dolphin	-2.07	0.30	0.30	6.83	0.00
Harbour porpoise	-1.40	0.25	0.26	5.47	0.00
Risso's dolphin	-1.38	0.44	0.44	3.14	0.00
Striped dolphin	-0.58	0.67	0.68	0.86	0.39
White-beaked dolphin	0.16	0.48	0.48	0.32	0.75
White-sided dolphin	-0.49	0.49	0.49	1.02	0.31
Latitude:Longitude	-0.08	0.06	0.06	1.31	0.19
Latitude:Days since 01/01/1990	0.20	0.04	0.04	4.56	0.00
Longitude:Days since 01/01/1990	-0.14	0.04	0.04	3.28	0.00
Days since 01/01/1990:Common dolphin	0.62	0.25	0.25	2.47	0.01
Days since 01/01/1990:Harbour porpoise	0.07	0.22	0.22	0.31	0.75
Days since 01/01/1990:Risso's dolphin	0.16	0.34	0.34	0.47	0.64
Days since 01/01/1990:Striped dolphin	0.35	0.50	0.50	0.70	0.48
Days since 01/01/1990:White-beaked dolphin	-0.28	0.37	0.37	0.77	0.44
Days since 01/01/1990:White-sided dolphin	0.07	0.42	0.42	0.17	0.86
Latitude:Longitude:Days since 01/01/1990	-0.01	0.03	0.03	0.32	0.75

Table S8: Model averaged coefficients for selected variables against blubber concentrations of HCHs.

	Estimate	Std. Error	Adjusted SE	z value	Pr(>|z|)
(Intercept)	-3.34	0.21	0.21	15.98	0.00
Age Group & Sex: AdultM	0.48	0.11	0.11	4.24	0.00
Age Group: Juvenile	0.56	0.09	0.09	6.06	0.00
Blubber thickness	-0.12	0.04	0.04	3.40	0.00
Latitude	0.00	0.04	0.04	0.02	0.98
Longitude	0.13	0.04	0.04	3.30	0.00
Days since 01/01/1990	-0.62	0.10	0.10	5.96	0.00
Common dolphin	0.01	0.24	0.25	0.04	0.97
Harbour porpoise	-0.30	0.20	0.20	1.50	0.13
Risso's dolphin	-0.23	0.33	0.33	0.71	0.48
Striped dolphin	0.40	0.39	0.39	1.01	0.31
White-beaked dolphin	0.52	0.34	0.34	1.53	0.13
White-sided dolphin	0.06	0.38	0.38	0.16	0.87
Latitude:Days since 01/01/1990	0.09	0.04	0.04	2.04	0.04
Longitude:Days since 01/01/1990	-0.13	0.04	0.04	3.40	0.00
Latitude:Longitude	0.00	0.03	0.03	0.19	0.85
Latitude:Longitude:Days since 01/01/1990	0.00	0.02	0.02	0.22	0.82
Days since 01/01/1990:Common dolphin	-0.01	0.08	0.08	0.15	0.88
Days since 01/01/1990:Harbour porpoise	-0.03	0.11	0.11	0.27	0.79
Days since 01/01/1990:Risso's dolphin	0.02	0.11	0.11	0.16	0.88
Days since 01/01/1990:Striped dolphin	0.00	0.14	0.14	0.02	0.98
Days since 01/01/1990:White-beaked dolphin	-0.03	0.13	0.13	0.20	0.84
Days since 01/01/1990:White-sided dolphin	-0.05	0.19	0.19	0.26	0.80

Table S9: Toxicity reference values (TRVs) used to derive Comparative Risk Quotients (CRQs) for each contaminant.

	Toxicity reference values (mg kg^{-1} day^{-1}))	
	Immunotoxicity	Endocrine disruption
PCBs	0.50	4.00
Dieldrin	0.65	0.50
DDTs	10.50	5.40
HCB	22.00	9.50
HCHs	20.00	0.50
PBDEs	0.13	10.00

† TRVs for each pollutant were taken from No Observed Adverse Effect Levels (NOAELS) from the Agency for Toxic Substances and Disease Registry Toxicological Profiles available at https://wwwn.cdc.gov/TSP/ToxProfiles/ToxProfiles.aspx?id=142&tid=26 [9–14]

Table S10: Comparative Risk Quotients (CRQs) for each pollutant and species, used to plot Figure 4.

Species	Pollutant	Comparitive Risk Quotient (Endocrine Disruption)	Comparitive Risk Quotient (Immunosuppression)
Bottlenose Dolphin	PCB	17.17	137.32
Bottlenose Dolphin	Dieldrin	1.31	1.01
Bottlenose Dolphin	DDTs	4.72	2.43
Bottlenose Dolphin	HCB	0.04	0.02
Bottlenose Dolphin	HCH	0.08	0.00
Bottlenose Dolphin	PBDEs	0.30	23.18
Common Dolphin	PCB	7.92	63.37
Common Dolphin	Dieldrin	0.96	0.74
Common Dolphin	DDTs	1.01	0.52
Common Dolphin	HCB	0.02	0.01
Common Dolphin	HCH	0.25	0.01
Common Dolphin	PBDEs	0.06	4.53
Common Seal	PCB	5.62	44.96
Common Seal	Dieldrin	0.06	0.04
Common Seal	DDTs	0.30	0.15
Common Seal	HCB	0.00	0.00
Common Seal	HCH	0.05	0.00

Common Seal	PBDEs	0.02	1.36
Grey Seal	PCB	1.59	12.72
Grey Seal	Dieldrin	0.08	0.06
Grey Seal	DDTs	0.26	0.13
Grey Seal	HCB	0.00	0.00
Grey Seal	HCH	0.06	0.00
Grey Seal	PBDEs	0.01	0.59
Harbour Porpoise	PCB	3.81	30.49
Harbour Porpoise	Dieldrin	1.56	1.20
Harbour Porpoise	DDTs	0.64	0.33
Harbour Porpoise	HCB	0.03	0.01
Harbour Porpoise	HCH	0.19	0.00
Harbour Porpoise	PBDEs	0.09	7.06
Killer Whale	PCB	84.17	673.37
Killer Whale	Dieldrin	41.23	31.72
Killer Whale	DDTs	55.07	28.32
Killer Whale	HCB	0.27	0.12
Killer Whale	HCH	0.33	0.01
Killer Whale	PBDEs	0.84	64.66
Risso's Dolphin	PCB	2.15	17.21
Risso's Dolphin	Dieldrin	0.44	0.34
Risso's Dolphin	DDTs	0.42	0.21
Risso's Dolphin	HCB	0.02	0.01
Risso's Dolphin	HCH	0.07	0.00
Risso's Dolphin	PBDEs	0.06	4.46
Striped Dolphin	PCB	9.59	76.68
Striped Dolphin	Dieldrin	1.11	0.85
Striped Dolphin	DDTs	3.28	1.68
Striped Dolphin	HCB	0.03	0.01
Striped Dolphin	HCH	0.13	0.00
Striped Dolphin	PBDEs	0.05	3.91
White-beaked Dolphin	PCB	8.65	69.22
White-beaked Dolphin	Dieldrin	1.79	1.37
White-beaked Dolphin	DDTs	2.27	1.17
White-beaked Dolphin	HCB	0.05	0.02
White-beaked Dolphin	HCH	0.14	0.00
White-beaked Dolphin	PBDEs	0.31	23.55
White-sided Dolphin	PCB	3.68	29.45

White-sided Dolphin	Dieldrin	1.78	1.37
White-sided Dolphin	DDTs	2.04	1.05
White-sided Dolphin	HCB	0.04	0.02
White-sided Dolphin	HCH	0.21	0.01
White-sided Dolphin	PBDEs	0.04	2.93

Table S11: Sample sizes of unpublished and previously published for each species and pollutants.

	PCBs		DDTs		Dieldrin		PBDEs		HCHs		HCB	
	New	Published	New	Published	New	Published	New	Published	New	Published	New	Published
Atlantic white-sided dolphin	22	0	12	0	12	0	11	0	12	0	22	0
Bottlenose dolphin	25	38 [15]	33	0	33	0	28	0	33	0	63	0
Common seal	16	0	9	0	9	0	9	0	9	0	16	0
Grey seal	21	0	7	0	7	0	7	0	7	0	21	0
Harbour porpoise	0	731[16]	169	489[17]	169	489[17]	5	415[17]	169	489[17]	247	489[17]
Killer whale	8	7[15]	10	0	10	0	8	0	10	0	15	0
Risso's dolphin	26	0	18	0	18	0	15	0	18	0	26	0
Short-beaked common dolphin	17	107[18]	80	0	80	0	10	0	80	0	125	0
Sperm whale	5[19]	1	5[19]	1	6	0	N/A	0	6	0	6	0
Striped dolphin	22	0	12	0	12	0	11	0	12	0	23	0
White-beaked dolphin	24	0	15	0	15	0	14	0	15	0	24	0

Table S12: Percentage of non-detects for each pollutant/congener

% of non-detects	Pollutant
26.65	CB.18
26.80	CB.28

36.37	CB.31
20.60	CB.44
6.36	CB.47
10.57	CB.49
2.99	CB.52
7.20	CB.66
0.31	CB.101
3.98	CB.105
12.86	CB.110
0.39	CB.118
0.61	CB.128
0.00	CB.138
15.70	CB.141
0.08	CB.149
3.68	CB.151
0.00	CB.153
5.90	CB.156
4.59	CB.158
0.31	CB.170
0.00	CB.180
0.92	CB.183
0.00	CB.187
4.13	CB.194
3.04	HCB
6.47	α-HCH
27.62	β- HCH
16.37	γ-HCH
1.22	DIELDRIN
0.00	ρρ-DDE’
0.00	ρρ-TDE’
0.16	ρρ-DDT’
52.84	BDE.17
33.73	BDE.28
0.18	BDE.47
33.50	BDE.66
66.03	BDE.85
4.29	BDE.99
2.36	BDE.100
66.36	BDE.138
9.44	BDE.153

9.09	BDE.154
49.33	BDE.183

Supplemental References List

1. Kannan, K., Blankenship, A.L., Jones, P.D., and Giesy, J.P. (2000). Toxicity reference values for the toxic effects of polychlorinated biphenyls to aquatic mammals. Hum. Ecol. Risk Assess. *6*, 181–201.
2. Desforges, J.-P., Sonne, C., Levin, M., Siebert, U., De Guise, S., and Dietz, R. (2016). Immunotoxic effects of environmental pollutants in marine mammals. Environ. Int. *86*, 126–139.
3. Hall, A.J., Kalantzi, O.I., and Thomas, G.O. (2003). Polybrominated diphenyl ethers (PBDEs) in grey seals during their first year of life - Are they thyroid hormone endocrine disrupters? Environ. Pollut. *126*, 29–37.
4. Lahvis, G.P., Wells, R.S., Kuehl, D.W., Stewart, J.L., Rhinehart, H.L., and Via, C.S. (1995). Decreased lymphocyte responses in free-ranging bottlenose dolphins (Tursiops truncatus) are associated with increased concentrations of PCBs and DDT in peripheral blood. Environ. Health Perspect. *103*, 67–72.
5. Stuart-Smith, S.J., and Jepson, P.D. (2017). Persistent threats need persistent counteraction: Responding to PCB pollution in marine mammals. Mar. Policy *84*, 69–75.
6. Pacyna, J.M., Breivik, K., Münch, J., and Fudala, J. (2003). European atmospheric emissions of selected persistent organic pollutants, 1970–1995. Atmos. Environ. *37*, 119–131.
7. Evenset, A., Christensen, G.N., Carroll, J., Zaborska, A., Berger, U., Herzke, D., and Gregor, D. (2007). Historical trends in persistent organic pollutants and metals recorded in sediment from Lake Ellasjøen, Bjørnøya, Norwegian Arctic. Environ. Pollut. *146*, 196–205.
8. Honeycutt, M., and Shirley, S. (2014). Dieldrin. In, P. B. T.-E. of T. (Third E. Wexler, ed. (Academic Press), pp. 107–110.
9. Agency for Toxic Substances and Disease Registry (ATSDR). (2022). Toxicological Profile for Aldrin and Dieldrin. Atlanta, GA: U.S. Department of Health and Human Services, Public Health Service.

https://wwwn.cdc.gov/TSP/ToxProfiles/ToxProfiles.aspx?id=317&tid=56.

10. Agency for Toxic Substances and Disease Registry (ATSDR) (2017). Toxicological profile for Polybrominated Diphenyl Ether (PBDEs). Atlanta,GA U.S. Dep. Heal. Hum. Serv. Public Heal. Serv. https://wwwn.cdc.gov/TSP/ToxProfiles/ToxProfiles.aspx?id=901&tid=183.

11. Agency for Toxic Substances and Disease Registry (ATSDR). (2022). Toxicological Profile for DDT, DDE, DDD. Atlanta, GA: U.S. Department of Health and Human Services, Public Health Service. https://wwwn.cdc.gov/TSP/ToxProfiles/ToxProfiles.aspx?id=81&tid=20.

12. Agency for Toxic Substances and Disease Registry (ATSDR) (2000). Toxicological profile for Polychlorinated Biphenyls (PCBs). Atlanta,GA U.S. Dep. Heal. Hum. Serv. Public Heal. Serv. https://www.atsdr.cdc.gov/toxprofiles/tp17.pdf.

13. Agency for Toxic Substances and Disease Registry (ATSDR) (2023). Toxicological Profile for Hexachlorocyclohexanes. Atlanta, GA: U.S. Department of Health and Human Services, Public Health Service. https://www.atsdr.cdc.gov/Toxprofiles/tp43.pdf.

14. Agency for Toxic Substances and Disease Registry (ATSDR) (2013). Toxicological profile for Hexachlorobenzene. GA U.S. Dep. Heal. Hum. Serv. Public Heal. Serv. https://www.atsdr.cdc.gov/toxprofiles/tp90.pdf.

15. Jepson, P.D., Deaville, R., Barber, J.L., Aguilar, À., Borrell, A., Murphy, S., Barry, J., Brownlow, A., Barnett, J., Berrow, S., et al. (2016). PCB pollution continues to impact populations of orcas and other dolphins in European waters. Sci. Rep. *6*, 18573.

16. Williams, R., ten Doeschate, M., Curnick, D.J., Brownlow, A., Barber, J.L., Davison, N.J., Deaville, R., Perkins, M., Jepson, P.D., and Jobling, S. (2020). Levels of polychlorinated biphenyls are still associated with toxic effects in harbor porpoises (Phocoena phocoena) despite having fallen below proposed toxicity thresholds. Environ. Sci. Technol. *54*, 2277–2286.

17. Law, R.J., Barry, J., Barber, J.L., Bersuder, P., Deaville, R., Reid, R.J., Brownlow, A., Penrose, R., Barnett, J., and Loveridge, J. (2012). Contaminants in cetaceans from UK waters: Status as assessed within the Cetacean Strandings Investigation Programme from 1990 to 2008. Mar. Pollut. Bull. *64*, 1485–1494.

18. Murphy, S., Law, R.J., Deaville, R., Barnett, J., Perkins, M.W., Brownlow, A., Penrose,

R., Davison, N.J., Barber, J.L., and Jepson, P.D. (2018). Organochlorine contaminants and reproductive implication in cetaceans: a case study of the common dolphin. Mar. mammal Ecotoxicol., 3–38.

19. Schnitzler, J.G., Pinzone, M., Autenrieth, M., van Neer, A., IJsseldijk, L.L., Barber, J.L., Deaville, R., Jepson, P., Brownlow, A., Schaffeld, T., et al. (2018). Inter-individual differences in contamination profiles as tracer of social group association in stranded sperm whales. Sci. Rep. *8*, 10958.
